# The listed, delisted, and sustainability of therapeutic medicines for dementia patients: the study is specific to South Korea

**DOI:** 10.1007/s00210-022-02209-3

**Published:** 2022-02-05

**Authors:** Jong Hoon Lee

**Affiliations:** Science & Research Center, Seoul National University College of Medicine, Seoul, South Korea

**Keywords:** Donepezil, Rivastigmine, Menantine, Mortality

## Abstract

The Dementia Management Act (DMA) came into effect on August 4, 2011, in South Korea. Diagnosis and medication were rapidly performed for dementia in a short time. We investigated the cardiac effects of increased drug prescription following DMA. We observed a correlation between Alzheimer’s disease (AD) and anti-AD drug (AAD) groups from 2010 to 2019 on the National Health Insurance System (NHIS) of South Korea. This study investigated the increase and decrease in deaths of AD patients with AAD. We analysed the mortality per 100,000 population with the *R*^2^ Calculator. Moreover, we made the up or down datum line for the simple decision on the listed, delisted, and sustainable drug examined by a linear equation and *R*^2^. We observed that life expectancy was diminished by AAD in Sorokdo National Hospital. In the NHIS, donepezil and rivastigmine increased the number of deaths decided on *R*^2^ > 0.75. Memantine was sustainable. We could not decide on galantamine because it is one of the other groups. We made a straightforward decision-maker of delisted, listed, or sustainable criteria based on mortality and datum line.

**Supplementary Information:**

The online version contains supplementary material available at 10.1007/s00210-022-02209-3.

## Introduction

National data about mortality in people with specific disabilities provide a basis for public health interventions. Life expectancy is data accumulated over decades. Linked mortality data using medical records to identify people with specific disabilities such as leprosy and intellectual disabilities could provide comprehensive, unbiased population‐based monitoring despite the circumstances of illnesses or death (Butlin 2020; Glover et al. 2017). For example, it is known that psychotropic medicines should not be administered to dementia patients because they increase mortality (Hunderfund et al. 2006; Stone 2005; Du et al. 1998).

The purpose of the Dementia Management Act (DMA) is to mitigate personal pain and damage from dementia. The DMA came into effect on August 04, 2011, in South Korea, and it was amended on June 12, 2018 (Management and Act,Act No.^15649^(2018).^2018^; Lee 2019a). As a result, medical personnel, psychiatrists, or neurologists of medical institutions and workers engaged in providing medical services under the Medical Service Act became very active in the dementia management programmes being executed by the state and local governments. According to the DMA policy, Alzheimer’s disease (AD) patients and anti-Alzheimer’s disease drug (AAD) prescriptions are increasing rapidly, so it is necessary to analyse national medical data. AAD group 1 received dementia symptom treatments: donepezil hydrochloride, rivastigmine, galantamine, and NMDA receptor antagonists. AAD group 2 included psychotropic medications such as haloperidol, risperidone, quetiapine, olanzapine, aripiprazole, oxcarbazepine, fluvoxamine, escitalopram, trazodone, sertraline, and fluoxetine.

South Korea’s electronic data interchange (EDI) medical procedure code is well computerised for health insurance claims data (Hwang et al. 2019). Therefore, general practitioners in South Korea currently identify patients with AD and taking AAD through EDI. This study investigated the changes in deaths and the AADs used to treat AD in the National Health Insurance System (NHIS) of South Korea. With the increasing medicalisation of dementia, we have announced that it can treat dementia or slow the disease’s progression (Greenberg et al. 2000; Seltzer et al. 2004; Tariot et al. 2004; Farlow and Cummings 2007; Raina et al. 2008; Lu et al. 2009; Narasimhalu et al. 2010). As a result, the prescriptions for AAD have increased dramatically, and it has been claimed that medications can treat dementia or slow disease progression.

Many toxins are cholinesterase inhibitors, and these toxins can cause death if given at high enough dosages. Cholinesterase inhibitors (ChEIs: donepezil, rivastigmine, galantamine) in patients with AD may affect heart rate, sometimes inducing bradycardia. There is no known cumulative effect on AD patients who have taken ChEIs or memantine consistently for long periods. C. E et al. reported QT prolongation, which is a measure of delayed ventricular repolarisation, and torsade de pointes with ChEIs: donepezil, rivastigmine, and galantamine in a sensitive whole-heart model of proarrhythmia. They observed that donepezil and galantamine amplified the spatial dispersion of repolarisation in rabbit hearts, but rivastigmine showed no increase in the spatial dispersion of repolarisation despite a pronounced prolongation of repolarisation. Furthermore, donepezil and galantamine provoked triggered activity, whereas rivastigmine did not have proarrhythmic effects (Ellermann et al. 2020; Isik et al. 2020). Rivastigmine’s crucial safety concern is the risk of treatment overdose by administering multiple patches simultaneously, potentially leading to fatal outcomes (Khoury et al. 2018; Kazmierski et al. 2018; Eijk et al. 2010). The promising theoretical mechanism of galantamine and donepezil seemed to be linked to nicotinic acetylcholine receptors (nAChRs), particularly α7 nAChR, but different for rivastigmine (Arias et al. 2005; Hoskin et al. 2018). Despite their promising effects complicated by adverse effects or minimal improvement, we analysed mortality by analysing all data on ChEIs or memantine based on NHIS big data.

## Methods

### Study design

The AAD First group is according to Korea Drug Code Medicine First Group: For symptomatic relief of Alzheimer’s disease (donepezil hydrochloride, rivastigmine, galantamine, N-methyl-D-aspartate (NMDA) receptor antagonist). The AAD Second group is according to Korea Drug Code Medicine Second Group: For psychologic symptoms of Alzheimer’s disease (haloperidol, risperidone, quetiapine, olanzapine, aripiprazole, oxcarbazepine, fluvoxamine, escitalopram, trazodone, sertraline, escitalopram, fluoxetine) (Youn and Jeong 2018). The mean age of death of AD patients was classified into the first or second group (Supplement S1).

We searched all medical records when the Korean government computerised the International Classification of Diseases (ICD)-10 code and Electronic Data Interchange (EDI) for the National Health Insurance System (NHIS). With the ICD-10 codes, medical data on the correlation between death and AAD were then analysed for age of death in the Sorokdo National Hospital, which is dedicated to Hansen’s disease (HD) patient care in South Korea.

Through the coordination of the Open Data Mediation Committee, data on the number of deaths amongst people taking AAD from 2010 to 2019 were available from the NHIS. We analysed the NHIS data of AAD and the deaths of all Koreans from 2010 to 2019. We used Object-Relational DBMS and Google spreadsheet for *R*^2^ analysis and linear equation and SPSS. We calculated the linear equation and *R*^2^ (0.75) for the increase, decrease, and indistinguishability.

### Ethics

The Korea National Institute for Bioethics Policy (KoNIBP) approved this study to manage life-sustaining treatment properly (approval number P01-202,007–22-006). The KoNIBP approved the observational study of patients ethically based on FDA guidelines following the World Medical Association Declaration of Helsinki.

### Population demography

HD patients have lived on Sorok Island for a lifetime. According to the request for disclosure of health checkup information from 2005 to 2020 on October 27, 2020, a total of 1321 people (694 males, 627 females) resided there, and the average age was 84.3 years (M 84.3, SD 17.1, 95% CI: 83.6–85.0).

National dementia demography consisted of inpatient, outpatient, and drug prescriptions with dementia ICD codes (F00, F01, F02, F03, G30, G31). Therefore, we analysed all Koreans with related ICD codes.

### Eligibility criteria

According to the Dementia Management Act, all Hansen subjects on Sorok Island are registered and treated at Sorokdo National Hospital. The Seoul cohort consisted of AD patients and AAD users in all Hansen subjects (Khattak et al. 2021). We searched all medical records of the Sorokdo National Hospital and NHIS in South Korea with ICD codes. The total number of persons with dementia eligible for national health insurance and medical benefits in South Korea from 2010 to 2019. In addition, we analysed medical data on the correlation between AD and AAD and death on the NHIS’s big data.

### Study setting

#### ICD code of Korean diseases and medicines

1. Mental and behavioural disorders, F00-F09, G30
  F00 code; Dementia in Alzheimer’s disease (G30.), F01 code; Vascular dementia, F02 code Dementia in other diseases classified elsewhere, F03 code; Unspecified dementia, F04 code; Organic amnesic syndrome, not induced by alcohol and other psychoactive substances, F05 code; Delirium, not induced by alcohol and other psychoactive substances, F06 code; Other mental disorders due to brain damage and dysfunction and to physical disease, F07 code; Personality and behavioural disorders due to brain disease, damage and dysfunction, F09 code; Unspecified organic or symptomatic mental disorder, G30 Alzheimer’s disease; (Supplement S2. Table S1.)
2. For symptomatic relief of Alzheimer’s disease
  First Group: For symptomatic relief of Alzheimer’s disease
  Donepezil hydrochloride; 148603ATB 148602ATD 148602ATB 148601ATD 148601ATB 643401ATD 643402ATD, rivastigmine; 224501ACH 224503ACH 224504ACH 224505ACH 224506CPC 224507CPC 224508CPC, galantamine; 385203ACR 385203ATR 385204ACR 385204ATR 385205ACR 385205ATR, N-methyl-D-aspartate (NMDA) receptor antagonist; 190031ALQ 190001ATB 190003ATD 190004ATB 190004ATD.
  Second Group: For psychologic symptoms of Alzheimer’s disease
  Haloperidol; 167903ATB 167904ATB 167905ATB 167906ATB 167908ATB 167908ATB 168030BIJ, Risperidone; 224201ATB 224201ATD 224202ATB 224202ATD 224203ATB 224204ATB 224205BIJ 224206BIJ, Quetiapine; 378601ATB 378602ATB 378603ATB 378604ATB 378605ATB 378605ATR 378606ATR 378607ATR 378608ATR 378608ATR 378610ATB, Olanzapine; 204001ATB 204001ATD 204002ATB 204002ATD 204004ATB 204005ATB, Aripiprazole; 451501ATB 451501ATD 451502ATB 451502ATD 451503ATB 451504ATB 451505ATB 451506BIJ 451507BIJ, Oxcarbazepine; 206330ASS 206301ATB 206302ATB 206303ATB, Fluvoxamine; 162501ATB 162502ATB, Escitalopram; 474801ATB 474802ATB 474803ATB 474804ATB, Trazodone; 242901ACH 242901ATB 242902ATB 242903ATR, Sertraline; 227001ATB 227002ATB 227003ATB, Escitalopram; 474801ATB 474802ATB 474803ATB 474804ATB, Fluoxetine; 161501ACH 161501ATB 161502ACH 161502ATB 161502ATD (Supplement S2. Table S2., S3.)
3. The number of users and deaths coded by the Open Data Mediation Committee

NHIS agreed to provide information following the Open Data Mediation Committee. 1486: donepezil hydrochloride, 2245: Rivastigmine, 1900: N-methyl-D-aspartate (NMDA) receptor antagonist, 2040: Olanzapine, 2242: Risperidone, 1615: Fluoxetine, 2270 Sertraline, 3786: Quetiapine, 4515 Aripiprazole, 4748 Escitalopram, 9999: The Others (Galantamine, Haloperidol, Fluvoxamine, Trazodone) (Supplement S2. Table S6.)

## Results

**Fig. 1:**
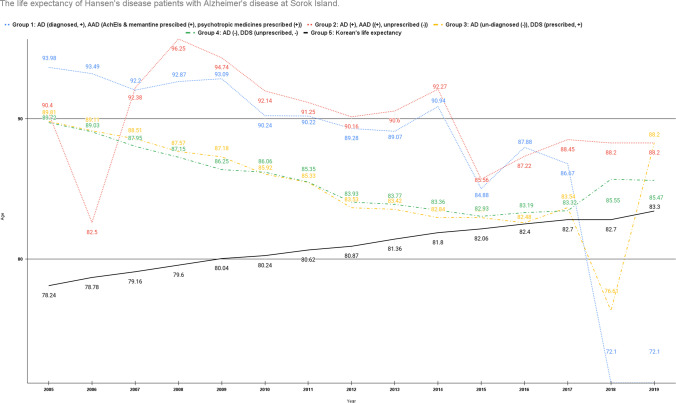
The expectancy of Hansen’s disease patients with Alzheimer’s disease at Sorok Island. In group 1 of Hansen’s disease (HD) patients with Alzheimer’s disease (AD) taking AChEIs or memantine with psychotropic medicines, the mean ages of death are blue. The mean ages of deaths without taking additional psychotropic drugs of group 2 are red. The life expectancy trends of HD patients taking other psychotropic medications (blue, group 1) were more decreased in the Sorokdo National Hospital. The life expectancies of group 3 (yellow, without AD, dapsone-prescribed) of HD patients or group 4 (green, without AD, dapsone-unprescribed) have been lower trends than group 1 or 2 before the year 2018–2019 (the period of Dementia National Responsibility System). Still, they all were above Korean’s life expectancy: group 5 (see black) until 2017. The life expectancy trends of HD patients taking anti-Alzheimer’s disease drug (AAD) were decreased on Sorok Island (blue, red), and those taking AAD with psychotropic medicines (blue) have been reduced more

Sorokdo National Hospital was established in May 1916 to treat leprosy. We connected the medical record database of the Sorokdo National Hospital and archived it from January 2005 to June 2019. With the ICD-10 codes, medical data on the correlation between DDS and AD were then analysed. In the group of patients diagnosed with Alzheimer’s disease, the average age of deaths while taking only drugs for dementia treatment (group 1) and the mean age of deaths while taking psychiatric drugs (group 1 and group 2) from 2005 to 2020 on October 27, a total of 2186 people (1152 males, 1034 females) resided there. The average age was 83.7 years (median (M) 84, interquartile range (IQR) 76.8–91.2, standard deviation (SD) 10.8, 95% confidence interval (CI): 0.45, 83.6–84.5). (Supplementary Tables S1–4) Compared with South Koreans’ life expectancy, there was a gradual decline in HD patients’ life expectancy with AD from 2005 to 2019 at Sorok Island. We observed that HD patients taking AAD group 1 and group 2 had a shorter lifespan than those taking AAD group 1 alone (Fig. 1).

**Fig. 2:**
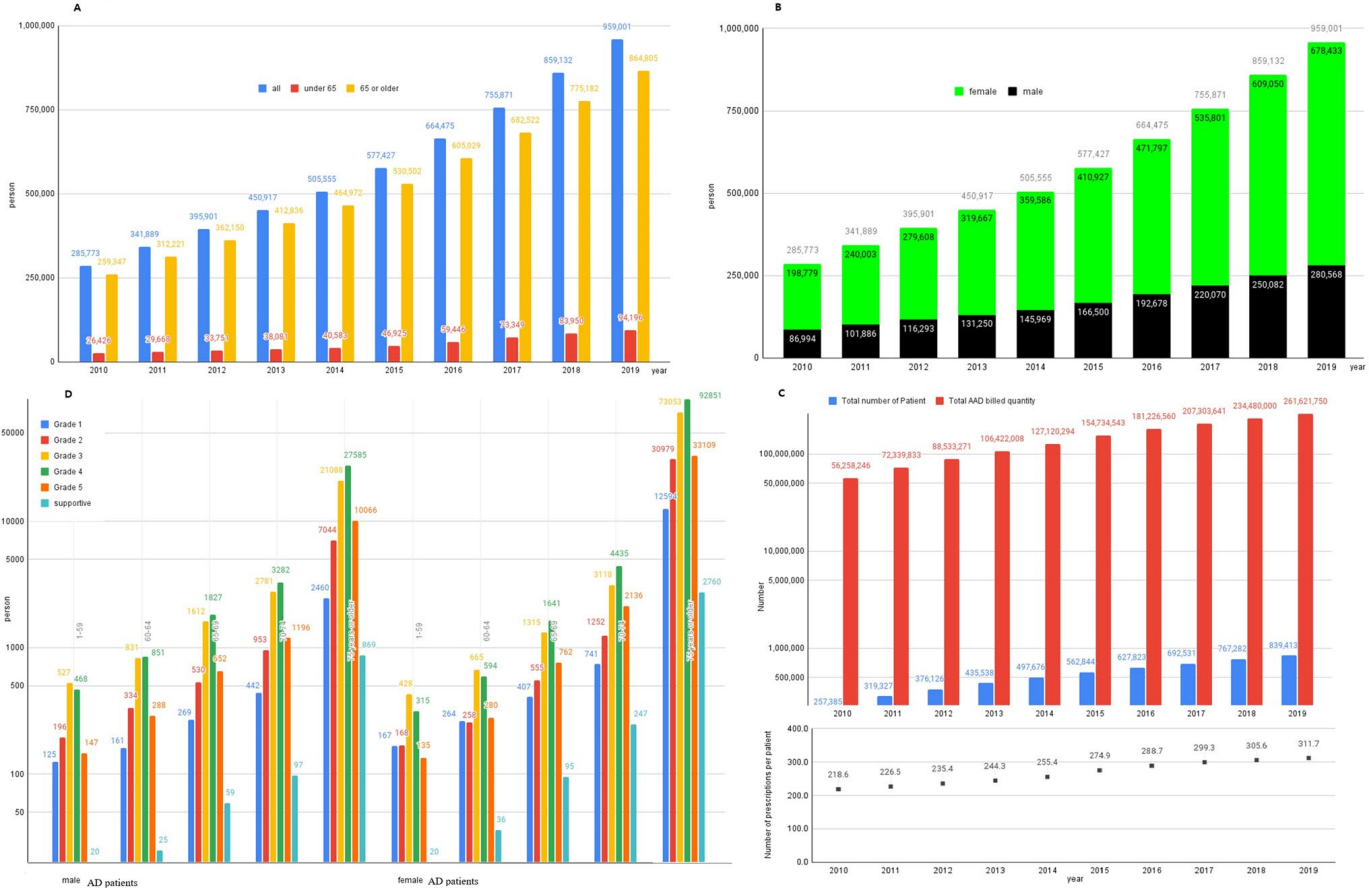
Demography of Korean dementia patients from 2010 to 2019. A^*^ From 2010 to 2019, the number of people with dementia has tripled over the past 10 years, faster than the growth rate of the population aged 65 years or older. In 2019, the estimated number of dementia patients aged 65 and over was approximately 790,000. B^*^ Women (62.9%) were higher than men. C^**^ From 2010 to June 2019, DMA increased the diagnosis of patients with mild cognitive impairment (MCI) or Alzheimer’s disease (AD) by 3.26 times and AAD prescription by 4.65 times in South Korea. As a result, the number of prescriptions per patient increased by 42.6%, from 218.6 to 311.7. D^*^ As of 2019, the mild dementia scale was supportive 1.2%, grade 5 13.8%, grade 4 38.0%, grade 3 29.9%, grade 2 12.0%, and grade 1 5.0% in 2019. (*This is the number of dementia patients reported to the Central Dementia Center of South Korea by the public health centre, which managed entire local residents. **This is the data diagnosed and prescribed at a medical institution and registered with the Health Insurance Review and Assessment Service of South Korea.)

In 2010, the number of elderly individuals over 65 was 5,348,182, and the number of people with dementia over 65 was 259,347 (4.8%). In 2019, the number of elderly individuals over 65 was 7,718,616, and the number of people with dementia over 65 years old was 864,805 (11.2%) who were diagnosed at a medical institution and registered with the Health Insurance Review and Assessment Service of South Korea. From 2010 to June 2019, DMA increased the diagnosis of patients with MCI or AD by 3.26 times and AAD prescription by 4.65 times in South Korea. The number of prescriptions per patient increased by 42.6%, from 218.6 to 311.7. As of 2019, the mild dementia scale was supportive 1.2%, grade 5 13.8%, grade 4 38.0%, grade 3 29.9%, grade 2 12.0%, and grade 1 5.0% in 2019. Grade 4 and grade 3, which required AAD medication for moderate dementia symptoms, accounted for 67.9%. Long-term care insurance in Korea provides services considering the functional status of difficulty in mobility by grade. Recipients of grades 1 and 2 are provided with a visiting service. The relatively mild grades 3 and 4 are designed to focus on day and night protection (4 times a week) considering the functional recovery training programme and social enhancement. The basic types such as dressing and washing face will be provided for grade 5 (Fig. 2).

We requested and analysed the entire ICD 10 code data (from 2010 to 2019) of AAD and deaths from the NHIS with the Open Data Mediation Committee of South Korea according to the Official Information Disclosure Act in South Korea. NHIS provided the information on deaths. However, galantamine, haloperidol, fluvoxamine, and trazodone were grouped as others to protect the confidentiality of pharmaceutical companies. The number of users who took AAD in Korea increased by 2.16 times, and the number of deaths increased by 2.51 times from 2010 to June 2019. The number of users who took donepezil increased by 3.48 times, and the number of deaths increased by 3.88 times. The number of users who took rivastigmine increased 1.84 times, and deaths increased by 2.36 times. The number of users who took memantine increased 2.50 times, and deaths increased by 2.29 times. The number of users who took risperidone increased 1.26 times, and deaths increased by 1.35 times. The number of users who took galantamine, haloperidol, fluvoxzmine, and trazodone (others) increased 1.55 times, and the number of deaths increased by 1.60 times from 2010 to June 2019 (Table 1). We adjusted for deaths per 100,000 population for comparison (Table 2).

**Table 1:** NHIS dementia medicines: users and deaths toll

Year	AAD		Donepezil		Rivastigmine		Memantine		Risperidone		Fluoxetine		Olanzapine		Sertraline		Quetiapine		Aripiprazole		Escitalopram		The others	
	User	Death	User	Death	User	Death	User	Death	User	Death	User	Death	User	Death	User	Death	User	Death	User	Death	User	Death	User	Death
2010	1,496,235	78,528	96,820	12,575	8,070	780	31,965	5,559	181,728	8945	156,899	1,802	37,810	1,654	71,494	1,341	140,218	10,654	25,155	330	227,186	5,093	518,890	29,795
2011	1,624,963	87,053	123,101	15,797	9,788	850	35,315	5,945	186,077	8976	151,252	1,580	45,177	2,001	76,809	1,319	172,218	13,466	28,561	382	258,981	5,949	537,684	30,788
2012	1,793,974	100,711	150,128	19,604	11,218	1,103	37,138	6,419	191,121	9519	150,654	1,582	54,344	2,681	85,193	1,377	202,486	17,654	37,290	466	303,698	7,060	570,704	33,246
2013	1,879,280	109,772	176,440	22,941	11,935	1,170	38,391	6,403	189,702	9354	140,194	1,439	59,184	3,112	86,931	1,427	228,140	21,117	45,297	570	326,645	7,585	576,421	34,654
2014	2,028,410	119,542	204,724	26,636	13,705	1,291	43,165	6,568	190,178	9271	134,013	1,395	63,444	3,276	89,114	1,565	259,635	24,520	67,152	768	359,582	8,118	603,698	36,134
2015	2,191,614	135,524	236,834	32,084	15,542	1,847	52,358	8,134	195,951	10,052	133,262	1,373	64,123	3,036	92,676	1,522	290,105	27,972	84,102	1,086	391,772	8,726	634,889	39,692
2016	2,373,538	148,351	267,241	36,375	15,103	1,682	58,626	9,080	199,776	10,396	137,846	1,329	66,902	3,368	103,099	1,684	331,811	32,972	102,241	1,164	431,465	9,298	659,428	41,003
2017	2,598,416	167,853	294,203	42,187	14,443	1,716	65,605	10,718	203,635	10,822	144,496	1,268	75,153	3,700	115,370	1,853	384,209	39,984	133,075	1,448	478,751	10,365	689,476	43,792
2018	2,880,654	185,099	319,751	47,487	14,777	1,839	70,873	11,597	208,994	11,179	157,170	1,369	81,868	4,042	129,710	1,982	439,704	46,257	174,861	1,895	549,235	11,480	733,711	45,972
2019	3,234,536	197,232	336,683	48,830	14,964	1,840	79,770	12,714	228,123	12,044	173,284	1,405	87,565	4,359	142,660	2,005	540,397	51,767	214,761	2,211	612,579	12,472	803,750	47,585

**Table 2:** Death rates per 100,000 population for calculation of NHIS dementia medicines

Deaths per 100,000 population												
Year	AAD	Donepezil	Rivastigmine	Memantine	Risperidone	Fluoxetine	Olanzapine	Sertaline	Quetiapine	Aripiprazole	Escitalopram	Others
2010	5248.4	12,988.0	9665.4	17,390.9	4922.2	1148.5	4374.5	1875.7	7598.2	1311.9	2241.8	5742.1
2011	5357.2	12,832.6	8684.1	16,834.2	4823.8	1044.6	4429.2	1717.2	7819.2	1337.5	2297.1	5726.0
2012	5613.8	13,058.2	9832.4	17,284.2	4980.6	1050.1	4933.4	1616.3	8718.6	1249.7	2324.7	5825.4
2013	5841.2	13,002.2	9803.1	16,678.4	4930.9	1026.4	5258.2	1641.5	9256.2	1258.4	2322.1	6011.9
2014	5893.4	13,010.7	9419.9	15,216.0	4874.9	1040.9	5163.6	1756.2	9444.0	1143.7	2257.6	5985.4
2015	6183.8	13,547.0	11,883.9	15,535.4	5129.9	1030.3	4734.7	1642.3	9642.0	1291.3	2227.3	6251.8
2016	6250.2	13,611.3	11,136.9	15,488.0	5203.8	964.1	5034.2	1633.4	9937.0	1138.5	2155.0	6218.0
2017	6459.8	14,339.4	11,881.2	16,337.2	5314.4	877.5	4923.3	1606.1	10,406.8	1088.1	2165.0	6351.5
2018	6425.6	14,851.2	12,445.0	16,363.1	5349.0	871.0	4937.2	1528.0	10,520.0	1083.7	2090.2	6265.7
2019	6097.7	14,503.3	12,296.2	15,938.3	5279.6	810.8	4978.0	1405.4	9579.4	1029.5	2036.0	5920.4

**Fig. 3:**
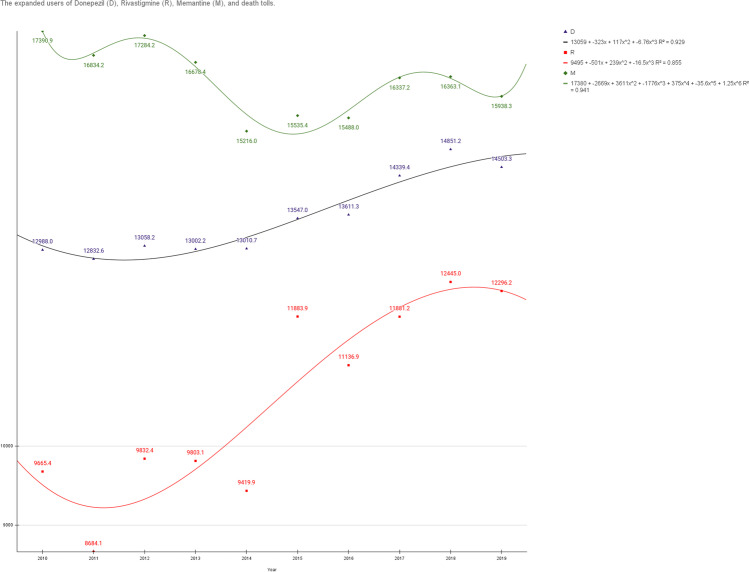
The expanded users of donepezil (D), rivastigmine (R), memantine (M), and death tolls. The donepezil trend line is black. Deaths per 100,000 people increase in the cubic polynomial equation (*R*^2^ = 0.929). The rivastigmine trend line is red. The death toll graph for rivastigmine increases rapidly from the year 2012 to the year 2018 in the cubic polynomial equation (*R*^2^ = 0.855). The memantine trend line is green. Memantine death tolls increase or decrease in the 6th-degree polynomial equation (*R*^2^ = 0.941)

The expanded use of donepezil increased the death toll, and rivastigmine increases the death toll more steeply from the year 2012 to the year 2018, which period was from the entry into force of the Dementia Management Act (2012) until the implementation of the National Dementia Responsibility System (2018). We reconfirmed that while the rivastigmine patch is easier to use than donepezil, it increases the risk of death. Memantine did not show a considerable increase in deaths compared with the expanded users (Fig. 3) (Supplement S2).

## Analysing mortality data for decision

**Fig. 4:**
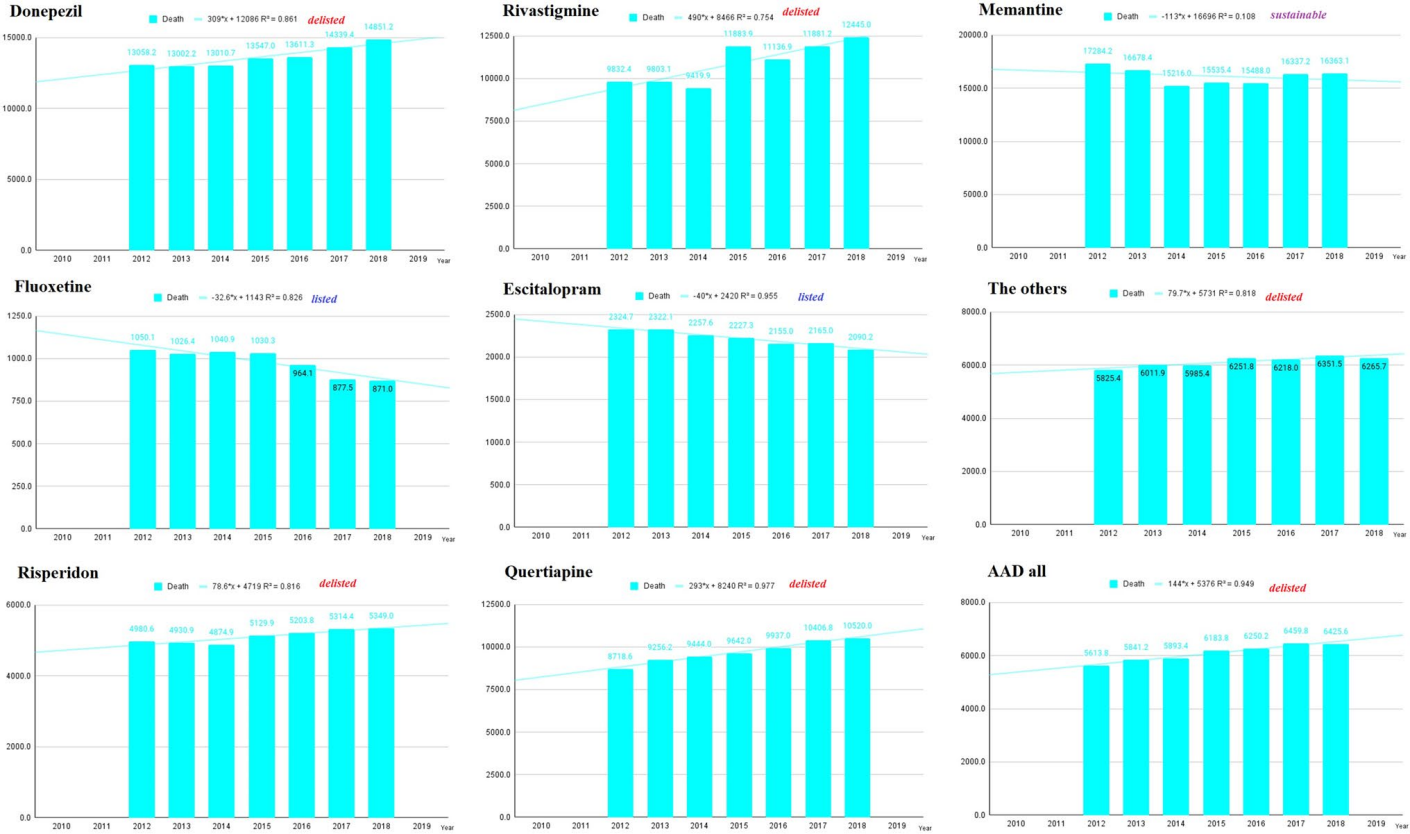
Linear equations and regression analysis from 2012 to 2018. The Dementia Management Act was enforced on February 05, 2012, and it was a partial amendment on June 12, 2018. We calculated the linear equation and *R*^2^. Donepezil, rivastigmine, risperidone, quetiapine, and others increased the number of deaths decided on *R*^2^ > 0.75. Fluoxetine and escitalopam decreased the number of deaths based on *R*^2^ > 0.75. Memantine was indistinguishable. ‘Delisted’, ‘Listed’, and ‘Sustainable’ are the simulation results of this study. ‘Delisted’ decision means the NHIS will no longer reimburse available symptomatic drugs against Alzheimer’s disease. ‘Sustainable’ means NHIS will observe whether to reimburse available symptomatic drugs against Alzheimer’s disease. ‘Listed’ means the NHIS will reimburse available symptomatic drugs against Alzheimer’s disease

DMA was enforced on February 05, 2012, and it was the partial amendment on June 12, 2018 (Management and Act,Act No.^15649^(2018). 2018). Therefore, we conducted an analysis of AAD and death for the period 2012–2018. We calculated the linear equation and *R*^2^. Donepezil, rivastigmine, risperidone, quetiapine, and others increased the number of deaths decided on *R*^2^ > 0.75. Fluoxetine and escitalopam decreased the number of deaths based on *R*^2^ > 0.75. Memantine, olanzapine, sertaline, and aripiprazone were indistinguishable (Fig. 4) (Supplement S3).

The NHIS and health care providers should not harm health care consumers (Lee et al. 2012). Therefore, we made the following decision: delisted (donepezil, rivastigmine, risperidone, quetiapine, and others), listed (fluoxetine and escitalopam), and sustainable (memantine, olanzapine, sertaline, and aripiprazone). ‘Delisted’ means that the NHIS will no longer reimburse available symptomatic drugs against Alzheimer’s disease. ‘Sustainable’ means NHIS will observe whether to reimburse available symptomatic drugs against Alzheimer’s disease (Krolak-Salmon et al. 2018). ‘Listed’ means the NHIS will reimburse available symptomatic drugs against Alzheimer’s disease (Table 3).

**Table 3:** The simulation of decision according to the results of *R*^2^ and formula

Code	Drug	Period	Formula	*R*^2^	Decision	Equation type
	AAD all	2012–2018	144*x + 5376	0.949	Delisted	Linear equation
**1486**	Donepezil	2012–2018	309*x + 12,086	0.861	Delisted^*^	Linear equation
**2245**	Rivastigmine	2012–2018	490*x + 8466	0.754	Delisted	Linear equation
**1900**	Memantine	2012–2018	− 113*x + 16,696	0.108	Sustainable^*^	Linear equation
**2242**	Risperidone	2012–2018	78.6*x + 4719	0.816	Delisted	Linear equation
**1802**	Fluoxetine	2012–2018	− 32.6*x + 1143	0.826	Listed^*^	Linear equation
**2040**	Olanzapine	2012–2018	− 28.1*x + 5138	0.124	Sustainable	Linear equation
**2270**	Sertaline	2012–2018	− 16.4*x + 1714	0.274	Sustainable	Linear equation
**3786**	Quetiapine	2012–2018	293*x + 8240	0.977	Delisted	Linear equation
**4515**	Aripiprazone	2012–2018	− 30.1*x + 1330	0.576	Sustainable	Linear equation
**4748**	Escitalopam	2012–2018	− 40*x + 2420	0.955	Listed	Linear equation
**9999**	The others	2012–2018	79.7*x + 5731	0.818	Delisted	Linear equation

^*^ ‘Delisted’ means the NHIS will no more reimburse available symptomatic drugs against Alzheimer’s disease. ^*^ ‘Sustainable’ means NHIS will observe whether to reimburse available symptomatic drugs against Alzheimer’s disease. ^*^ ‘Listed’ means the NHIS will reimburse available symptomatic drugs against Alzheimer’s disease

## Discussion

The initial study on the longevity of dementia patients was conducted from 2005 to 2020 based on the medical records of Sorokdo National Hospital. Sister Marianne Stoeger and Sister Margaritha Pissarek served with exceptional compassion for their patients from February 1962 to November 21, 2005. However, the average life expectancy of Hansen’s disease (HD) patients began to decline overall in the four groups in 2005. The life expectancy of HD patients was estimated to be longer than that of Koreans. Hypotheses were proposed because HD patients had been taking dapsone (Cho et al. June 2014; Cho et al. 2010, 2011; Park 2017; Choi et al. 2019). Even more embarrassing, the expectancy of taking AAD was higher than that of the group not taking AAD, and all four groups tended to decrease. The groups on Sorok Island show that death tolls are more critical when studying AAD. Dapsone has been proven to protect HD patients as an inflammasome competitor from diverse inflammasome-induced diseases (Lee et al. 2020a). This study studied only the number of deaths. The reason is that the number of deaths is the most apparent and essential data that can measure the effect of treatment. S. B et al. reported that donepezil initiation was associated with better survival benefits than memantine and oral and transdermal forms of rivastigmine from AD medication groups in a US national sample of Medicare beneficiaries (Bhattacharjee et al. 2019). Rivastigmine is dangerous to AD patient survival, according to the results of our study. The AD2000 Collaborative Group’s research reported in 2004 that donepezil is not cost-effective, with benefits below minimally relevant thresholds. There were no significant results between donepezil and placebo in adverse events or deaths, formal care costs, unpaid caregiver time, carer psychopathology, or behavioural and psychological symptoms. There was also no significant difference between 5 and 10 mg donepezil. More effective treatments than cholinesterase inhibitors are needed to treat AD (Courtney et al. 2004). ChEIs and memantine did not reduce the progression rate of Alzheimer’s disease (Petersen et al. 2005; Feldman et al. 2007; Raschetti et al. 2007; Dysken et al. 2014; Winblad et al. 2008). AD patients who received ChEIs and memantine took them for longer, were more functionally impaired, and showed more significant cognitive decline than those who only received ChEIs (Schneider et al. 2011). When assessing the hazard of death in persons with or without amnestic mild cognitive impairment (MCI), MCI is associated with increased mortality (Hunderfund et al. 2006). The French Minister of Health published a decree on May 29, 2018, removing the drugs (donepezil, rivastigmine, galantamine, and memantine) used to fight against symptoms due to Alzheimer’s disease from the list of available reimbursed drugs (Krolak-Salmon et al. 2018). However, in our study, donepezil and rivastigmine were delisted while memantine was sustainable.

## Korean government’s legislative process and AAD medication

The Korean government has established national policies for dementia care, and compulsory long-term care insurance for the elderly was introduced (Chon 2014). The ‘War against Dementia’ and the First National Dementia Plan were announced in 2008 (Lee 2019a). It facilitates the socialisation of long-term care services at a national level. The DMA was legislated in August 2011. The government announced the DMA as a reform plan, emphasising changes such as increasing coverage and improving the quality of services (Chon 2014). The DMA intended to lighten children’s burden and help enhance national health by establishing and implementing comprehensive policies on preventing dementia, supporting dementia patients, and researching a cure for dementia. However, the DMA reinforced the socialisation of elder care, and the enduring fear of dependency in old age forced Koreans to actively cooperate in diagnostic tests and treatments for dementia (Lee 2019b).

As a result of the election in May 2017, the new president announced the National Duty for Dementia. The DMA was strengthened on June 12, 2018. The Korean government successively installed Community Dementia Reassurance Centers at all Community Health Centers to establish a community-based dementia management system according to the National Duty for Dementia. Psychiatrists or neurologists of medical institutions engaged in medical diagnosis and treatment under the Medical Service Act (Youn and Jeong 2018). They strengthened the dementia management programmes that administer AAD to MCI or AD patients as a preventive and treatment (Lee et al. 2009; Ahn et al. 2015). They insisted that the 1-year persistence rate of ChEIs for AD patients should be specially monitored to optimise treatment persistence because patients are less likely to remain on therapy than those in other countries (Ahn et al. 2015). However, the researchers already published the results of no improvement in 2005–2009 (Feldman et al. 2007; Winblad et al. 2008).

Nonetheless, the medical staff published ChEIs and Memantine as significant, modest therapeutic improvements in 2009 (Lee et al. 2009; Kang et al. 2012). By Article 12 (1) of the DMA, the government and local governments provided support for the treatment and diagnosis of dementia in consideration of the economic burden of dementia patients. The NHIS reimbursed the cost of AAD, and the drugs became almost free. From 2010 to June 2019, policymakers and medical staff increased patients to take AAD by 2.16 times and die by 2.51 times in South Korea and perhaps worldwide.

## The neurological side effects of ChEIs for AD patients

The percentage of new users was 2.5% across hospitalisations for AD medication (Möllers et al. 2020). Neuropsychiatric symptoms and adverse drug reactions were associated with a significantly increased prevalence of further psychotropic medication use (Stingl et al. 2020). Hospital stays due to dementia and the need for care were predictors for the new use of psychotropic medication (Jordan et al. 2019). All studies from many countries have already confirmed that antipsychotic drugs should not be administered to dementia patients because of the risk of seizures and all-cause mortality (Stone 2005; Du et al. 1998). J. N and T. D reported the feasibility of arrhythmia monitoring using an implantable loop recorder and the incidence of arrhythmia in a population of psychiatric outpatients receiving psychotropic medication (Nordgaard and Melchior 2021). Patients with mental disorders have an increased risk of premature death compared with the background population. Many of these drugs affect the heart’s conduction system and lead to life-threatening ventricular arrhythmias (Nordgaard and Melchior 2021).

The neurological side effects of ChEIs are similar to the neurological symptoms of AD patients. Only specialists can distinguish the side effects caused by donepezil or dementia symptoms, such as dizziness, delusions, dream abnormalities, ataxia, convulsive seizures, hemiplegia, hypertonia, and salivation (Mendez et al. 2007; Lee et al. 2020b; Lee et al. 2021). J. L reported that clinicians should record donepezil as a biomarker (D) according to the Alzheimer’s continuum (A + T + [N] + (D)) and plotted the radial chart to prove its usefulness for monitoring (D)’s side effects after neurosurgeon’s prescribing acetylcholine precursor according to the National Institute on Aging (NIA) at the National Institutes of Health and the Alzheimer’s Association (NIA-AA)’s revised guidelines for modernisation of the diagnosis of Alzheimer’s disease (Lee et al. 2021). While monitoring the AD patient’s condition, acetylcholine precursor was prescribed in the hospital’s intensive care unit to the patient who had a stroke. However, medical staff were unaware of the acetylcholine precursor’s side effects caused by the neurological symptoms (Lee et al. 2021).

## Many toxins are cholinesterase inhibitors

There was no known cumulative effect on AD patients who had taken ChEIs or memantine consistently for long periods before this study. Acetylcholine performs various physiologic functions through cholinergic muscarinic receptors: five different muscarinic receptors, M1, M2, M3, M4, and M5. The muscarinic receptor M2 is present in smooth muscle and cardiac tissue (Birdsall et al. 1988; Lebois et al. 2018). Many toxins are cholinesterase inhibitors, and they cause death if given at high dosages. Botulinum toxin blocks the release of acetylcholine hormone from the presynaptic terminal by preventing acetylcholine release (Huang et al. 2000). Black widow spider venom is thought to be associated with a wide release of neurotransmitters, especially norepinephrine and acetylcholine, due to spider envenomation. It causes the cells to release acetylcholine, which stimulates excessive muscle contractions. Paralysis occurs if widow venom exhausts all acetylcholine supplies as the opposite effect of botulinum toxin (Tzeng et al. 1978; Yan and Wang 2015).

## Limitations of our study

We are fully aware of the limitations of our study. We studied just one country within one dementia field and a limited time. Future studies should aim to analyse other countries and more extended periods. From a broader perspective, other biomedical research areas and clinical specialities should, of course, also be analysed.

## Conclusion

We suggested a straightforward decision-maker of delisted or listed or sustainable criteria based on mortality and datum line.

## The more data, the better

Despite this work’s inherent limitations and problems, we hope this study provides a suitable template for an objective analysis of dementia patients’ care in biomedical science. As always in science, the more data we have, the better conclusions we can draw and act accordingly.

## Supplementary Information

Below is the link to the electronic supplementary material.

Supplementary file1 (DOCX 1009 KB)

## Data Availability

The author declares that all primary data generated or analysed during this study supporting the findings are available in the article and supplement files. Additional data that support the findings of this study are available from the corresponding author upon reasonable request. Additional data are provided at the Center for Open Science (Lee J. Death Toll by Dementia Drug [Internet]. OSF; 2021. Available from: osf.io/z7ph2).
