# Supplementary material for: The listed, delisted, and sustainability of therapeutic medicines for dementia patients: the study is specific to South Korea
Source: Naunyn Schmiedebergs Arch Pharmacol. 2022 Feb 5;395(5):535–46. doi: 10.1007/s00210-022-02209-3 (PMC8989833; doi:10.1007/s00210-022-02209-3)
Supplement: Supplementary file 1 — Supplementary file1 (DOCX 1009 KB) [file 210_2022_2209_MOESM1_ESM.docx]

**Supplementary Material for**

**The Listed, Delisted, and Sustainability of the therapeutic medicines for Dementia patients**

Jong-Hoon Lee

**Correspondence to:** science@research.re.kr

The National Agency approved this study for Management of Life-sustaining Treatment, which certified that the life-sustaining treatments were managed properly (Korea National Institute for Bioethics Policy (KoNIBP) approval number P01-202007-22-006).

According to the Official Information Disclosure Act in Korea, the Seoul study analyzed AD and anti-Alzheimer's disease drug (AAD) use in Hansen subjects. We searched all medical records of the National Health Insurance Service (NHIS) in Korea and the Sorokdo National Hospital from the time when the International Classification of Diseases (ICD)-10 code and Electronic Data Interchange (EDI) were computerized.

The Sorokdo National Hospital was established in May 1916 to treat leprosy. We connected to the medical record database of the Sorokdo National Hospital and archived it from January 2005 to June 2020. With the ICD-9 and -10 codes, medical data on the correlation between DDS and AD were then analyzed. In the group of patients diagnosed with Alzheimer's disease, the average age of deaths while taking only drugs for dementia treatment (group 1) and the mean age of deaths with taking psychiatric drugs (group 1 & group 2).

According to the Official Information Disclosure Act in Korea, we also requested and analyzed the entire ICD 10 code data (from 2010 to 2019) of AD and AAD from the Health Insurance Review & Assessment system. From 2010 to June 2020, the diagnosis of patients with MCI and AD in Korea increased dramatically.

Contents

[Supplement Section (S) 1. Korea Drug Code Medicine 5](#_Toc84936179)

[Supplement Section 2. NHIS Dementia Data of South Korea 10](#_Toc84936180)

[Supplement Section 3. Analysis 16](#_Toc84936181)

[Supplement Section 4. Statistics 45](#_Toc84936182)

[Supplement Section 5. Dementia Demography of NHIS from Central Dementia Center. 48](#_Toc84936183)

Tables

[Table S1. Mental and Behavioural Disorders, F00-F09, G30 5](#_Toc93246197)

[Table S2. First Group: For Symptomatic Relief of Alzheimer’s Disease 5](#_Toc93246198)

[Table S3. Second Group: For Psychologic Symptoms of Alzheimer’s Disease 5](#_Toc93246199)

[Table S4. The Life expectancy of Hansen's disease patients in the Sorokdo National Hospital 6](#_Toc93246200)

[Table S5. Numbers of drug prescriptions for dementia patients in South Korea from 2010 to June 2019 10](#_Toc93246201)

[Table S6-1. Numbers of dementia patients in South Korea from 2010 to 2019 10](#_Toc93246202)

[Table S7-2. Grades of dementia patients in South Korea from 2010 to 2019 11](#_Toc93246203)

[Table S8. NHIS Masked Code and ICD-9, -10 Code 14](#_Toc93246204)

[Table S9. Anti-Alzheimer's drug (AAD) and User and the Death Toll 16](#_Toc93246205)

[Table S10. Donepezil code and User and the Death Toll 18](#_Toc93246206)

[Table S11. Rivastigmine and User and Death Toll 21](#_Toc93246207)

[Table S12. Memantine and User and the Death Toll 24](#_Toc93246208)

[Table S13. Risperidone and User and Death Toll 27](#_Toc93246209)

[Table S14. Fluoxetine and User and Death Toll 30](#_Toc93246210)

[Table S15. Olanzapine and User and Death Toll 32](#_Toc93246211)

[Table S16. Sertaline and User and Death Toll 34](#_Toc93246212)

[Table S17. Quetiapine and User and Death Toll 36](#_Toc93246213)

[Table S18. Aripiprazone and User and Death Toll 38](#_Toc93246214)

[Table S19. Escitalopram and User and the Death Toll 40](#_Toc93246215)

[Table S20. The others and User and the Death Toll 42](#_Toc93246216)

[Table S21. NHIS Dementia Medicines: Users and Deaths Toll 45](#_Toc93246217)

[Table S22. NHIS Dementia Medicines: Users and Deaths Toll 47](#_Toc93246218)

Figures

[Fig. S1. Numbers of drug prescriptions for dementia patients in Korea from 2010 to 2019. 13](#_Toc93246219)

[Fig. S2. AAD Trend line. 17](#_Toc93246220)

[Fig. S3. Donepezil Trend line. 19](#_Toc93246221)

[Fig. S4. Rivastigmine Trend line. 3.8E-03*x + 0.133 R^2^ = 0.006 22](#_Toc93246222)

[Fig. S5. Memantine Trend line. 25](#_Toc93246223)

[Fig. S6. Risperidone Trend line. 28](#_Toc93246224)

[Fig. S7. Fluoxetine Trend line. 31](#_Toc93246225)

[Fig. S8. Olanzapine Trend line. 33](#_Toc93246226)

[Fig. S9. Sertaline Trend line. 35](#_Toc93246227)

[Fig. S10. Quetiapine Trend line. 37](#_Toc93246228)

[Fig. S11. Aripiprazone Trend line. 39](#_Toc93246229)

[Fig. S12. Escitalopram Trend line. 41](#_Toc93246230)

[Fig. S13. AAD The others line. 43](#_Toc93246231)

Supplement Section (S) 1. Korea Drug Code Medicine

Korean Standard Classification of Diseases (KCD) code for "Alzheimer's disease."

(1) KCD code for Alzheimer's disease.

Table S1. Mental and Behavioural Disorders, F00-F09, G30

| F00 code | Dementia in Alzheimer's disease (G30.-+) |
| --- | --- |
| F01 code | Vascular dementia |
| F02 code | Dementia in other diseases classified elsewhere |
| F03 code | Unspecified dementia |
| F04 code | Organic amnesic syndrome, not induced by alcohol and other psychoactive substances |
| F05 code | Delirium, not induced by alcohol and other psychoactive substances |
| F06 code | Other mental disorders due to brain damage and dysfunction and to physical disease |
| F07 code | Personality and behavioural disorders due to brain disease, damage and dysfunction |
| F09 code | Unspecified organic or symptomatic mental disorder |
| G30 code | Alzheimer's disease |

(2) For symptomatic relief of Alzheimer's disease

Table S2. First Group: For Symptomatic Relief of Alzheimer’s Disease

| donepezil hydrochloride | 148603ATB 148602ATD 148602ATB 148601ATD 148601ATB 643401ATD 643402ATD |
| --- | --- |
| rivastigmine | 224501ACH 224503ACH 224504ACH 224505ACH 224506CPC 224507CPC 224508CPC |
| galantamine | 385203ACR 385203ATR 385204ACR 385204ATR 385205ACR 385205ATR |
| N-methyl-D-aspartate (NMDA) receptor antagonist (Memantine) | 190031ALQ 190001ATB 190003ATD 190004ATB 190004ATD |

Table S3. Second Group: For Psychologic Symptoms of Alzheimer’s Disease

| haloperidol | 167903ATB 167904ATB 167905ATB 167906ATB 167908ATB 167908ATB 168030BIJ |
| --- | --- |
| Risperidone | 224201ATB 224201ATD 224202ATB 224202ATD 224203ATB 224204ATB 224205BIJ 224206BIJ |
| Quetiapine | 378601ATB 378602ATB 378603ATB 378604ATB 378605ATB 378605ATR 378606ATR 378607ATR 378608ATR 378608ATR 378610ATB |
| Olanzapine | 204001ATB 204001ATD 204002ATB 204002ATD 204004ATB 204005ATB |
| Aripiprazole | 451501ATB 451501ATD 451502ATB 451502ATD 451503ATB 451504ATB 451505ATB 451506BIJ 451507BIJ |
| Oxcarbazepine | 206330ASS 206301ATB 206302ATB 206303ATB |
| fluvoxamine | 162501ATB 162502ATB |
| Escitalopram | 474801ATB 474802ATB 474803ATB 474804ATB |
| Trazodone | 242901ACH 242901ATB 242902ATB 242903ATR |
| Sertraline | 227001ATB 227002ATB 227003ATB |
| Escitalopram | 474801ATB 474802ATB 474803ATB 474804ATB |
| Fluoxetine | 161501ACH 161501ATB 161502ACH 161502ATB 161502ATD |

(2) HD patients in Sorok Island with KCD code for Alzheimer's disease.

Table S4. The Life expectancy of Hansen's disease patients in the Sorokdo National Hospital

| year | AD First group & Second group ^1^ | AD First group without Second group^2^ | AD (-) & DDS (+) group^3^ | AD (-) & DDS (-) group^4^ | The life expectancy of Korean |
| --- | --- | --- | --- | --- | --- |
| 2005 | 93.98 | 90.4 | 89.81 | 89.72 | 78.24 |
| 2006 | 93.49 | 82.5 | 89.11 | 89.03 | 78.78 |
| 2007 | 92.2 | 92.38 | 88.51 | 87.95 | 79.16 |
| 2008 | 92.87 | 96.25 | 87.57 | 87.15 | 79.6 |
| 2009 | 93.09 | 94.74 | 87.18 | 86.25 | 80.04 |
| 2010 | 90.24 | 92.14 | 85.92 | 86.06 | 80.24 |
| 2011 | 90.22 | 91.25 | 85.33 | 85.35 | 80.62 |
| 2012 | 89.28 | 90.16 | 83.53 | 83.93 | 80.87 |
| 2013 | 89.07 | 90.6 | 83.42 | 83.77 | 81.36 |
| 2014 | 90.94 | 92.27 | 82.84 | 83.36 | 81.8 |
| 2015 | 84.88 | 85.56 | 82.84 | 82.93 | 82.06 |
| 2016 | 87.88 | 87.22 | 82.48 | 83.19 | 82.4 |
| 2017 | 86.67 | 88.45 | 83.54 | 83.32 | 82.7 |
| 2018 | 72.1 | 88.2 | 76.61 | 85.55 | 82.7 |
| 2019 | 72.1 | 88.2 | 88.2 | 85.47 | 83.3 |

^1^ Group 1: AD (diagnosed, +), AAD (AchEIs & memantine prescibed, (+), psychotropic medicines unprescribed (+))^, 2^ Group 2: AD (+), AAD (+, -)), ^3^  Group 3: AD (un-diagnosed (-)), DDS (prescribed, +), ^4^ Group 4: AD (-), DDS (-)

**Population demography of Sorok Island.**

HD patients have lived on Sorok Island for a lifetime. According to the request for disclosure of health checkup information from 2005 to 2020 on October 27, 2020, a total of 2186 people (1152 males, 1034 females) resided there, and the average age was 83.7 years (median (M) 84, interquartile range (IQR) 76.8 – 91.2, standard deviation (SD) 10.8, 95% confidence interval (CI): 0.45, 83.6 – 84.5).

- Reference: Study of AD group in Sorok Island (1)

Participants were randomized in a 2:1 ratio to DDS (+) or matching placebo DDS (-). Randomization was unrestricted (no blocking or stratification), and we analyzed the ICE codes in databases of Sorokdo National Hospital. A 2:1 allocation ratio was done because of HD patients taking dapsone for a lifetime. Therefore, it increases participants' probability of receiving the active study drug without compromising statistical power. Furthermore, the 2:1 allocation ratio led to a 10% increase in overall sample size relative to a 1:1 allocation ratio(2).

Calculation: STUDY OF AD (+/-) GROUP

Supplementary Table 1. AD (+) Prevalence in the dapsone (+/-) subgroup.

| year | DDS (+) | DDS (-) | sum AD (+) | Mean | SD | 95% CI | [CI | CI] |
| --- | --- | --- | --- | --- | --- | --- | --- | --- |
| 2005 | 18 | 19 | 37 | 18.5 | 0.71 | 0.24 | 18.26 | 19.21 |
| 2006 | 20 | 37 | 57 | 28.5 | 12.02 | 3.19 | 25.31 | 40.52 |
| 2007 | 22 | 51 | 73 | 36.5 | 20.51 | 4.78 | 31.72 | 57.01 |
| 2008 | 22 | 58 | 80 | 40 | 25.46 | 5.66 | 34.34 | 65.46 |
| 2009 | 19 | 66 | 85 | 42.5 | 33.23 | 7.17 | 35.33 | 75.73 |
| 2010 | 25 | 82 | 107 | 53.5 | 40.31 | 7.73 | 45.77 | 93.81 |
| 2011 | 35 | 98 | 133 | 66.5 | 44.55 | 7.64 | 58.86 | 111.05 |
| 2012 | 39 | 135 | 174 | 87 | 67.88 | 10.16 | 76.84 | 154.88 |
| 2013 | 34 | 172 | 206 | 103 | 97.58 | 13.40 | 89.60 | 200.58 |
| 2014 | 25 | 190 | 215 | 107.5 | 116.67 | 15.68 | 91.82 | 224.17 |
| 2015 | 26 | 242 | 268 | 134 | 152.74 | 18.37 | 115.63 | 286.74 |
| 2016 | 33 | 255 | 288 | 144 | 156.98 | 18.21 | 125.79 | 300.98 |
| 2017 | 37 | 268 | 305 | 152.5 | 163.34 | 18.40 | 134.10 | 315.84 |
| 2018 | 45 | 292 | 337 | 168.5 | 174.66 | 18.71 | 149.79 | 343.16 |
| 2019 | 46 | 334 | 380 | 190 | 203.65 | 20.54 | 169.46 | 393.65 |
| 2020 | 32 | 352 | 384 | 192 | 226.27 | 22.70 | 169.30 | 418.27 |
| Sum | 478 | 2651 | 3129 |  |  |  |  |  |
| Mean | 29.88 | 165.69 | 195.56 |  |  |  |  |  |
| SD | 9.03 | 112.31 | 119.15 |  |  |  |  |  |
| 95% CI | 0.81 | 4.28 | 4.18 |  |  |  |  |  |
|  | 29.06 | 161.41 | 191.39 |  |  |  |  |  |
|  | 30.69 | 169.96 | 199.74 |  |  |  |  |  |

Supplementary Table 2. AD (-) Prevalence in the dapsone (+/-) subgroup.

| year | DDS (+) | DDS (-) | sum AD (-) | Mean | SD | 95% CI | [CI | CI] |
| --- | --- | --- | --- | --- | --- | --- | --- | --- |
| 2005 | 290 | 417 | 707 | 353.5 | 89.80 | 6.63 | 346.87 | 443.30 |
| 2006 | 302 | 363 | 665 | 332.5 | 43.13 | 3.28 | 329.22 | 375.63 |
| 2007 | 317 | 332 | 649 | 324.5 | 10.61 | 0.82 | 323.68 | 335.11 |
| 2008 | 310 | 312 | 622 | 311 | 1.41 | 0.11 | 310.89 | 312.41 |
| 2009 | 300 | 283 | 583 | 291.5 | 12.02 | 0.98 | 290.52 | 303.52 |
| 2010 | 270 | 286 | 556 | 278 | 11.31 | 0.94 | 277.06 | 289.31 |
| 2011 | 255 | 268 | 523 | 261.5 | 9.19 | 0.79 | 260.71 | 270.69 |
| 2012 | 238 | 241 | 479 | 239.5 | 2.12 | 0.19 | 239.31 | 241.62 |
| 2013 | 195 | 248 | 443 | 221.5 | 37.48 | 3.50 | 218.00 | 258.98 |
| 2014 | 172 | 236 | 408 | 204 | 45.25 | 4.40 | 199.60 | 249.25 |
| 2015 | 167 | 168 | 335 | 167.5 | 0.71 | 0.08 | 167.42 | 168.21 |
| 2016 | 154 | 149 | 303 | 151.5 | 3.54 | 0.40 | 151.10 | 155.04 |
| 2017 | 143 | 115 | 258 | 129 | 19.80 | 2.43 | 126.57 | 148.80 |
| 2018 | 132 | 87 | 219 | 109.5 | 31.82 | 4.24 | 105.26 | 141.32 |
| 2019 | 114 | 40 | 154 | 77 | 52.33 | 8.33 | 68.67 | 129.33 |
| 2020 | 109 | 4 | 113 | 56.5 | 74.25 | 13.84 | 42.66 | 130.75 |
| Sum | 3468 | 3549 |  |  |  |  |  |  |
| Mean | 216.75 | 221.81 |  |  |  |  |  |  |
| SD | 76.04 | 117.71 |  |  |  |  |  |  |
| 95% CI | 2.53 | 3.87 | 4.45 |  |  |  |  |  |
|  | 214.22 | 217.94 | 434.11 |  |  |  |  |  |
|  | 219.28 | 225.69 | 443.01 |  |  |  |  |  |

Supplementary Table 3. AD Prevalence in the Dapsone (+/-) subgroup.

| Year | DDS (+) | DDS (-) | AD (+) | Dapsone (+) | Dapsone (-) | AD (-) | P-value* | chi-square | p-value |
| --- | --- | --- | --- | --- | --- | --- | --- | --- | --- |
| 2005 | 18 | 19 | 37 | 290 | 417 | 707 | .3583 | 0.8438 | 0.358314 |
| 2006 | 20 | 37 | 57 | 302 | 363 | 665 | .1324 | 2.2655 | 0.132282 |
| 2007 | 22 | 51 | 73 | 317 | 332 | 649 | .0024** | 9.22 | 0.002394 |
| 2008 | 22 | 58 | 80 | 310 | 312 | 622 | .00028** | 14.191 | 0.000165 |
| 2009 | 19 | 66 | 85 | 300 | 283 | 583 | < .00001** | 25.1874 | 0.00001 |
| 2010 | 25 | 82 | 107 | 270 | 286 | 556 | < .00001** | 23.0669 | 0.00001 |
| 2011 | 35 | 98 | 133 | 255 | 268 | 523 | < .00001** | 21.651 | 0.00001 |
| 2012 | 39 | 135 | 174 | 238 | 241 | 479 | < .00001** | 38.8685 | 0.00001 |
| 2013 | 34 | 172 | 206 | 195 | 248 | 443 | < .00001** | 46.6137 | 0.00001 |
| 2014 | 25 | 190 | 215 | 172 | 236 | 408 | < .00001** | 60.6925 | 0.00001 |
| 2015 | 26 | 242 | 268 | 167 | 168 | 335 | < .00001** | 110.2835 | 0.00001 |
| 2016 | 33 | 255 | 288 | 154 | 149 | 303 | < .00001** | 105.7934 | 0.00001 |
| 2017 | 37 | 268 | 305 | 143 | 115 | 258 | < .00001** | 120.4582 | 0.00001 |
| 2018 | 45 | 292 | 337 | 132 | 87 | 219 | < .00001** | 134.6692 | 0.00001 |
| 2019 | 46 | 334 | 380 | 114 | 40 | 154 | < .00001** | 200.2285 | 0.00001 |
| 2020 | 32 | 352 | 384 | 109 | 4 | 113 | < .00001** | 333.6671 | 0.00001 |
|  |  |  |  |  |  |  |  | 252.58 | < 0.00001 |

Supplement Section 2. NHIS Dementia Data of South Korea

Table S5. Numbers of drug prescriptions for dementia patients in South Korea from 2010 to June 2019

| Year | Quantity of drugs | Amount billed for drugs | Number of patients | Number of prescriptions per patient |
| --- | --- | --- | --- | --- |
| 2,010 | 56,258,246 | 109,447,005 | 257,385 | 218.6 |
| 2,011 | 72,339,833 | 134,113,061 | 319,327 | 226.5 |
| 2,012 | 88,533,271 | 140,102,940 | 376,126 | 235.4 |
| 2,013 | 106,422,008 | 160,202,046 | 435,538 | 244.3 |
| 2,014 | 127,120,294 | 187,253,891 | 497,676 | 255.4 |
| 2,015 | 154,734,543 | 215,276,824 | 562,844 | 274.9 |
| 2,016 | 181,226,560 | 240,267,891 | 627,823 | 288.7 |
| 2,017 | 207,303,641 | 265,949,724 | 692,531 | 299.3 |
| 2,018 | 234,480,000 | 296,443,092 | 767,282 | 305.6 |
| 2,019 | 261,621,750 | 328,425,771 | 839,413 | 311.7 |

(Currency data provided by Morningstar on February 7, 12:58 AM UTC)

▶ Source: National Health Insurance Corporation (2010-2019)

▶ Extraction criteria :

A person with dementia has been assigned a total of six dementia codes (F00, F01, F02, F03, G30, G31). (Dementia treatment: Inpatient, outpatient, and drug prescription with dementia code assigned as main/injury code.) The status of the total number of persons with dementia who are eligible for health insurance and eligible for medical benefits, changes in qualifications are not reflected. There is a slight difference in the number of persons with dementia.

Table S6-1. Numbers of dementia patients in South Korea from 2010 to 2019

( Unit: person )

| year |  | 2010 | 2011 | 2012 | 2013 | 2014 | 2015 | 2016 | 2017 | 2018 | 2019 |
| --- | --- | --- | --- | --- | --- | --- | --- | --- | --- | --- | --- |
| gender | age group |  |  |  |  |  |  |  |  |  |  |
|  | all | 285,773 | 341,889 | 395,901 | 450,917 | 505,555 | 577,427 | 664,475 | 755,871 | 859,132 | 959,001 |
|  | under 60 | 13,388 | 15,179 | 17,718 | 20,293 | 21,434 | 24,114 | 30,168 | 37,453 | 40,624 | 42,812 |
|  | 60 or older | 272,385 | 326,710 | 378,183 | 430,624 | 484,121 | 553,313 | 634,307 | 718,418 | 818,508 | 916,189 |
|  | under 65 | 26,426 | 29,668 | 33,751 | 38,081 | 40,583 | 46,925 | 59,446 | 73,349 | 83,950 | 94,196 |
|  | 65 or older | 259,347 | 312,221 | 362,150 | 412,836 | 464,972 | 530,502 | 605,029 | 682,522 | 775,182 | 864,805 |
| total | 40-44 | 871 | 845 | 975 | 1,081 | 1,003 | 1,032 | 1,243 | 1,340 | 1,410 | 1,345 |
|  | 45-49 | 1,778 | 1,854 | 2,050 | 2,366 | 2,247 | 2,487 | 3,059 | 3,679 | 3,784 | 3,752 |
|  | 50-54 | 4,179 | 4,569 | 5,360 | 5,930 | 6,026 | 6,493 | 7,895 | 9,577 | 10,198 | 11,045 |
|  | 55-59 | 6,560 | 7,911 | 9,333 | 10,916 | 12,158 | 14,102 | 17,971 | 22,857 | 25,232 | 26,670 |
|  | 60-64 | 13,038 | 14,489 | 16,033 | 17,788 | 19,149 | 22,811 | 29,278 | 35,896 | 43,326 | 51,384 |
|  | 65-69 | 25,822 | 28,477 | 29,317 | 32,019 | 35,334 | 41,401 | 49,050 | 55,789 | 65,183 | 75,238 |
|  | 70-74 | 46,545 | 54,193 | 62,462 | 68,110 | 71,641 | 78,055 | 84,228 | 87,329 | 97,844 | 110,037 |
|  | 75-79 | 63,159 | 76,221 | 87,800 | 100,172 | 111,144 | 122,742 | 137,466 | 155,853 | 172,229 | 183,020 |
|  | 80-84 | 61,583 | 75,299 | 88,301 | 100,770 | 115,608 | 134,774 | 156,337 | 175,339 | 199,795 | 222,812 |
|  | 85-89 | 41,961 | 51,528 | 60,759 | 70,658 | 83,802 | 97,973 | 113,031 | 130,807 | 149,474 | 169,975 |
|  | 90-94 | 15,996 | 20,886 | 26,429 | 32,818 | 37,591 | 43,894 | 50,524 | 59,281 | 68,610 | 79,724 |
|  | 95-99 | 3,713 | 4,871 | 6,108 | 7,154 | 8,539 | 10,096 | 12,457 | 15,782 | 19,445 | 21,027 |
|  | over 100 | 568 | 746 | 974 | 1,135 | 1,313 | 1,567 | 1936 | 2,342 | 2,602 | 2,972 |
| Male (M) | 40-44 | 473 | 475 | 516 | 559 | 478 | 513 | 584 | 635 | 655 | 572 |
|  | 45-49 | 969 | 1006 | 1,041 | 1,167 | 1,077 | 1,163 | 1,327 | 1,548 | 1,520 | 1,461 |
|  | 50-54 | 2,088 | 2,294 | 2,415 | 2,554 | 2,582 | 2,726 | 3,204 | 3,621 | 3,631 | 3,861 |
|  | 55-59 | 3,208 | 3,794 | 4,157 | 4,693 | 5,194 | 5,827 | 7,072 | 8,679 | 8,990 | 9,154 |
|  | 60-64 | 6,159 | 6,638 | 7,000 | 7,419 | 7,993 | 9,165 | 11,512 | 14,000 | 16,088 | 18,393 |
|  | 65-69 | 10,261 | 11,382 | 11,671 | 12,707 | 14,169 | 16,530 | 19,372 | 21,617 | 24,344 | 27,509 |
|  | 70-74 | 16,436 | 19,062 | 22,095 | 24,060 | 25,210 | 27,679 | 30,349 | 32,061 | 35,968 | 40,676 |
|  | 75-79 | 19,122 | 23,001 | 26,685 | 30,924 | 34,506 | 38,289 | 43,540 | 50,237 | 55,925 | 60,569 |
|  | 80-84 | 15,417 | 18,401 | 21,836 | 25,107 | 29,175 | 35,154 | 41,675 | 47,402 | 55,285 | 63,085 |
|  | 85-89 | 9,230 | 11,104 | 12,936 | 14,878 | 17,306 | 19,918 | 23,097 | 27,452 | 32,556 | 37,928 |
|  | 90-94 | 3,017 | 3,946 | 4,950 | 6,023 | 6,861 | 7,782 | 8,796 | 10,121 | 11,839 | 13,835 |
|  | 95-99 | 537 | 684 | 867 | 1,024 | 1,272 | 1,576 | 1,939 | 2,438 | 2,968 | 3,141 |
|  | over 100 | 77 | 99 | 124 | 135 | 146 | 178 | 211 | 259 | 313 | 384 |
| M total |  | 86,994 | 101,886 | 116,293 | 131,250 | 145,969 | 166,500 | 192,678 | 220,070 | 250,082 | 280,568 |
| % |  | 30.4 | 29.8 | 29.4 | 29.1 | 28.9 | 28.8 | 29.0 | 29.1 | 29.1 | 29.3 |
| Female (F) | 40-44 | 398 | 370 | 459 | 522 | 525 | 519 | 659 | 705 | 755 | 773 |
|  | 45-49 | 809 | 848 | 1,009 | 1,199 | 1,170 | 1,324 | 1,732 | 2,131 | 2,264 | 2,291 |
|  | 50-54 | 2,091 | 2,275 | 2,945 | 3,376 | 3,444 | 3,767 | 4,691 | 5,956 | 6,567 | 7,184 |
|  | 55-59 | 3,352 | 4,117 | 5,176 | 6,223 | 6,964 | 8,275 | 10,899 | 14,178 | 16,242 | 17,516 |
|  | 60-64 | 6,879 | 7,851 | 9,033 | 10,369 | 11,156 | 13,646 | 17,766 | 21,896 | 27,238 | 32,991 |
|  | 65-69 | 15,561 | 17,095 | 17,646 | 19,312 | 21,165 | 24,871 | 29,678 | 34,172 | 40,839 | 47,729 |
|  | 70-74 | 30,109 | 35,131 | 40,367 | 44,050 | 46,431 | 50,376 | 53,879 | 55,268 | 61,876 | 69,361 |
|  | 75-79 | 44,037 | 53,220 | 61,115 | 69,248 | 76,638 | 84,453 | 93,926 | 105,616 | 116,304 | 122,451 |
|  | 80-84 | 46,166 | 56,898 | 66,465 | 75,663 | 86,433 | 99,620 | 114,662 | 127,937 | 144,510 | 159,727 |
|  | 85-89 | 32,731 | 40,424 | 47,823 | 55,780 | 66,496 | 78,055 | 89,934 | 103,355 | 116,918 | 132,047 |
|  | 90-94 | 12,979 | 16,940 | 21,479 | 26,795 | 30,730 | 36,112 | 41,728 | 49,160 | 56,771 | 65,889 |
|  | 95-99 | 3,176 | 4,187 | 5,241 | 6,130 | 7,267 | 8,520 | 10,518 | 13,344 | 16,477 | 17,886 |
|  | over 100 | 491 | 647 | 850 | 1,000 | 1,167 | 1,389 | 1,725 | 2,083 | 2,289 | 2,588 |
| F total |  | 198,779 | 240,003 | 279,608 | 319,667 | 359,586 | 410,927 | 471,797 | 535,801 | 609,050 | 678,433 |
| % |  | 69.9 | 70.2 | 70.6 | 70.9 | 71.1 | 71.2 | 71.0 | 70.9 | 70.9 | 70.7 |

Table S7-2. Grades of dementia patients in South Korea from 2010 to 2019

( Unit: person )

|  |  |  | Grade 1 | Grade 2 | Grade 3 | Grade 4 | Grade 5 | supportive | total |
| --- | --- | --- | --- | --- | --- | --- | --- | --- | --- |
|  |  |  |  |  |  |  |  |  | 352,165 |
| Nationwide | male | 1-59 | 125 | 196 | 527 | 468 | 147 | 20 | 1,483 |
|  |  | 60-64 | 161 | 334 | 831 | 851 | 288 | 25 | 2490 |
|  |  | 65-69 | 269 | 530 | 1,612 | 1,827 | 652 | 59 | 4,949 |
|  |  | 70-74 | 442 | 953 | 2,781 | 3,282 | 1,196 | 97 | 8,751 |
|  |  | 75 years or older | 2,460 | 7,044 | 21,088 | 27,585 | 10,066 | 869 | 69,112 |
|  | female | 1-59 | 167 | 168 | 428 | 315 | 135 | 20 | 1,233 |
|  |  | 60-64 | 264 | 258 | 665 | 594 | 280 | 36 | 2,097 |
|  |  | 65-69 | 407 | 555 | 1,315 | 1,641 | 762 | 95 | 4,775 |
|  |  | 70-74 | 741 | 1,252 | 3,118 | 4,435 | 2,136 | 247 | 11,929 |
|  |  | 75 years or older | 12,594 | 30,979 | 73,053 | 92,851 | 33,109 | 2,760 | 245,346 |


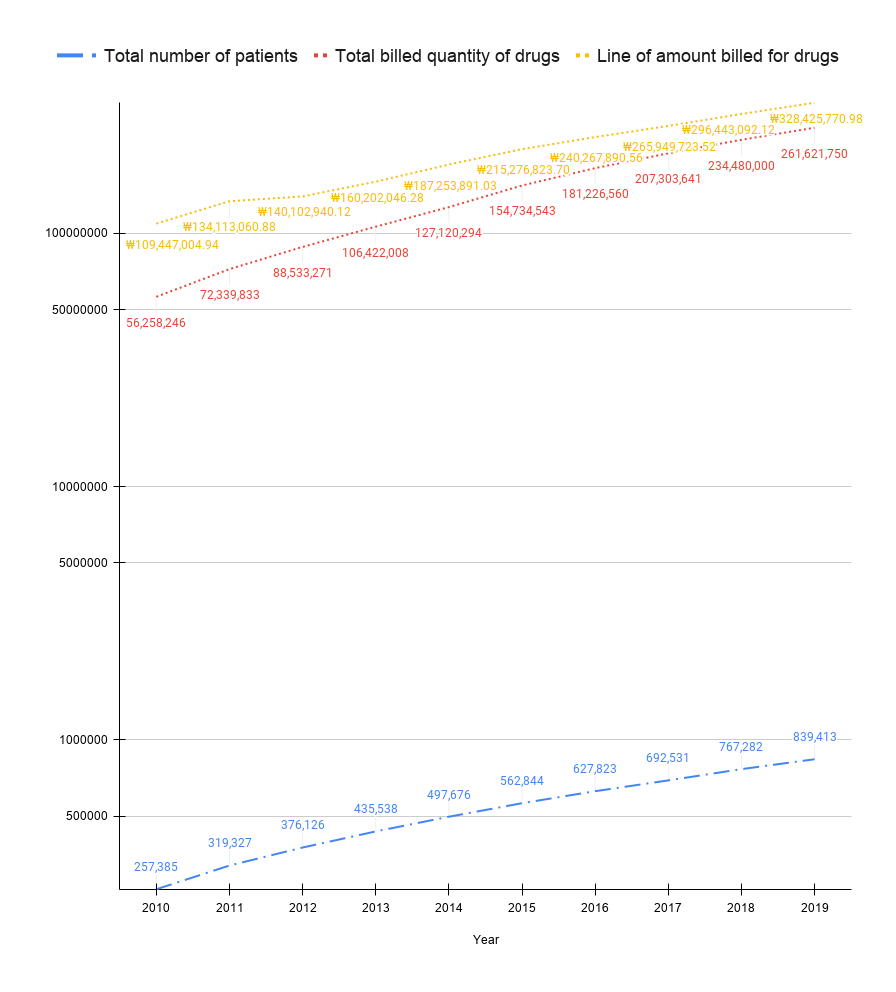


Fig. S1. Numbers of drug prescriptions for dementia patients in Korea from 2010 to 2019.

The state or local government subsidizes dementia patients for expenses incurred in the treatment and diagnosis of dementia from its budget, considering each dementia patient's capability to bear such costs. The AD and AAD data were reported from the Health Insurance Review & Assessment system. From 2010 to June 2020, the diagnosis and prescription of patients with MCI and AD in Korea increased 3.26 times and 4.65 times, respectively.

NHIS refused to provide information according to the Official Information Disclosure Act in Korea. The reason was that there was an obligation to protect the business information of pharmaceutical companies. So, the Open Data Mediation Committee should do its best to provide a more efficient and prompt dispute mediation service, intervening in the dispute between NHIS and researchers. Eventually, it was agreed to provide the number of users and deaths for code like this. We decided to accept it and draw a regression graph to analyze the correlation between the Dementia Management Act trends in 2018 through regression (R^2^ ) analysis.

Table S8. NHIS Masked Code and ICD-9, -10 Code

| Masked code | ICD 9, 10 code | Drug |
| --- | --- | --- |
| 1486 | 148603ATB 148602ATD 148602ATB 148601ATD 148601ATB 643401ATD 643402ATD | donepezil hydrochloride |
| 2245 | 224501ACH 224503ACH 224504ACH 224505ACH 224506CPC 224507CPC 224508CPC | Rivastigmine |
| 1900 | 190031ALQ 190001ATB 190003ATD 190004ATB 190004ATD | N-methyl-D-aspartate (NMDA) receptor antagonist |
| 2040 | 204001ATB 204001ATD 204002ATB 204002ATD 204004ATB 204005ATB | Olanzapine |
| 2242 | 224201ATB 224201ATD 224202ATB 224202ATD 224203ATB 224204ATB 224205BIJ 224206BIJ | Risperidone |
| 1615 | 161501ACH 161501ATB 161502ACH 161502ATB 161502ATD | Fluoxetine |
| 2270 | 227001ATB 227002ATB 227003ATB | Sertraline |
| 3786 | 378601ATB 378602ATB 378603ATB 378604ATB 378605ATB 378605ATR 378606ATR 378607ATR 378608ATR 378608ATR 378610ATB | Quetiapine |
| 4515 | 451501ATB 451501ATD 451502ATB 451502ATD 451503ATB 451504ATB 451505ATB 451506BIJ 451507BIJ | Aripiprazole |
| 4748 | 474801ATB 474802ATB 474803ATB 474804ATB | Escitalopram |
| 9999 | Galantamine, Haloperidol, Fluvoxamine, Trazodone  385203ACR 385203ATR 385204ACR 385204ATR 385205ACR 385205ATR  167903ATB 167904ATB 167905ATB 167906ATB 167908ATB 167908ATB 168030BIJ  162501ATB 162502ATB  242901ACH 242901ATB 242902ATB 242903ATR | The Others |

# Supplement Section 3. Analysis

Table S9. Anti-Alzheimer's drug (AAD) and User and the Death Toll

| Year | AAD | AAD Users | Death |
| --- | --- | --- | --- |
| 2010 | all | 1496235 | 78528 |
| 2011 | all | 1624963 | 87053 |
| 2012 | all | 1793974 | 100711 |
| 2013 | all | 1879280 | 109772 |
| 2014 | all | 2028410 | 119542 |
| 2015 | all | 2191614 | 135524 |
| 2016 | all | 2373538 | 148351 |
| 2017 | all | 2598416 | 167853 |
| 2018 | all | 2880654 | 185099 |
| 2019 | all | 3234536 | 197232 |

| Year | AAD | AAD Users | Death |
| --- | --- | --- | --- |
| 2010 | all | 100000 | 5248.4 |
| 2011 | all | 100000 | 5357.2 |
| 2012 | all | 100000 | 5613.8 |
| 2013 | all | 100000 | 5841.2 |
| 2014 | all | 100000 | 5893.4 |
| 2015 | all | 100000 | 6183.8 |
| 2016 | all | 100000 | 6250.2 |
| 2017 | all | 100000 | 6459.8 |
| 2018 | all | 100000 | 6425.6 |
| 2019 | all | 100000 | 6097.7 |

(Deaths per 100,000 population)


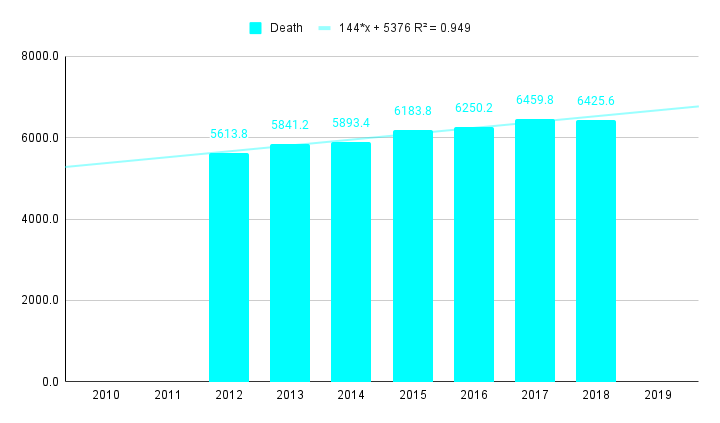

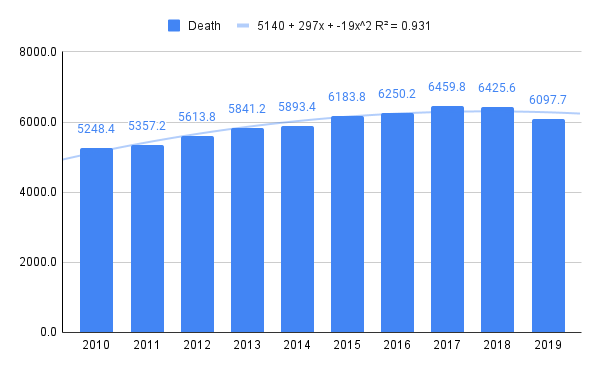


Fig. S2. AAD Trend line.

Table S10. Donepezil code and User and the Death Toll

| Year | Donepezil | Donepezil Users | Death |
| --- | --- | --- | --- |
| 2010 | 1486 | 96,820 | 12,575 |
| 2011 | 1486 | 123,101 | 15,797 |
| 2012 | 1486 | 150,128 | 19,604 |
| 2013 | 1486 | 176,440 | 22,941 |
| 2014 | 1486 | 204,724 | 26,636 |
| 2015 | 1486 | 236,834 | 32,084 |
| 2016 | 1486 | 267,241 | 36,375 |
| 2017 | 1486 | 294,203 | 42,187 |
| 2018 | 1486 | 319,751 | 47,487 |
| 2019 | 1486 | 336,683 | 48,830 |

| Year | Donepezil | Donepezil Users | Death |
| --- | --- | --- | --- |
| 2010 | 1486 | 100000 | 12988.0 |
| 2011 | 1486 | 100000 | 12832.6 |
| 2012 | 1486 | 100000 | 13058.2 |
| 2013 | 1486 | 100000 | 13002.2 |
| 2014 | 1486 | 100000 | 13010.7 |
| 2015 | 1486 | 100000 | 13547.0 |
| 2016 | 1486 | 100000 | 13611.3 |
| 2017 | 1486 | 100000 | 14339.4 |
| 2018 | 1486 | 100000 | 14851.2 |
| 2019 | 1486 | 100000 | 14503.3 |

(Deaths per 100,000 population)


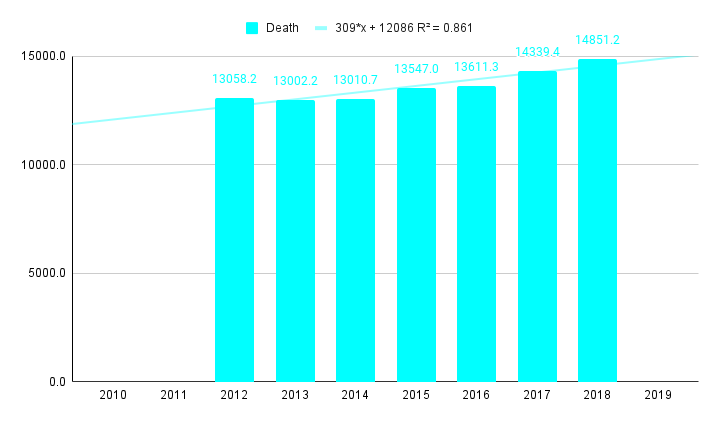

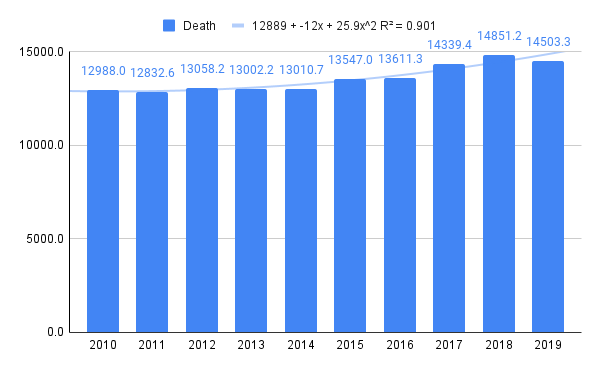


Fig. S3. Donepezil Trend line.


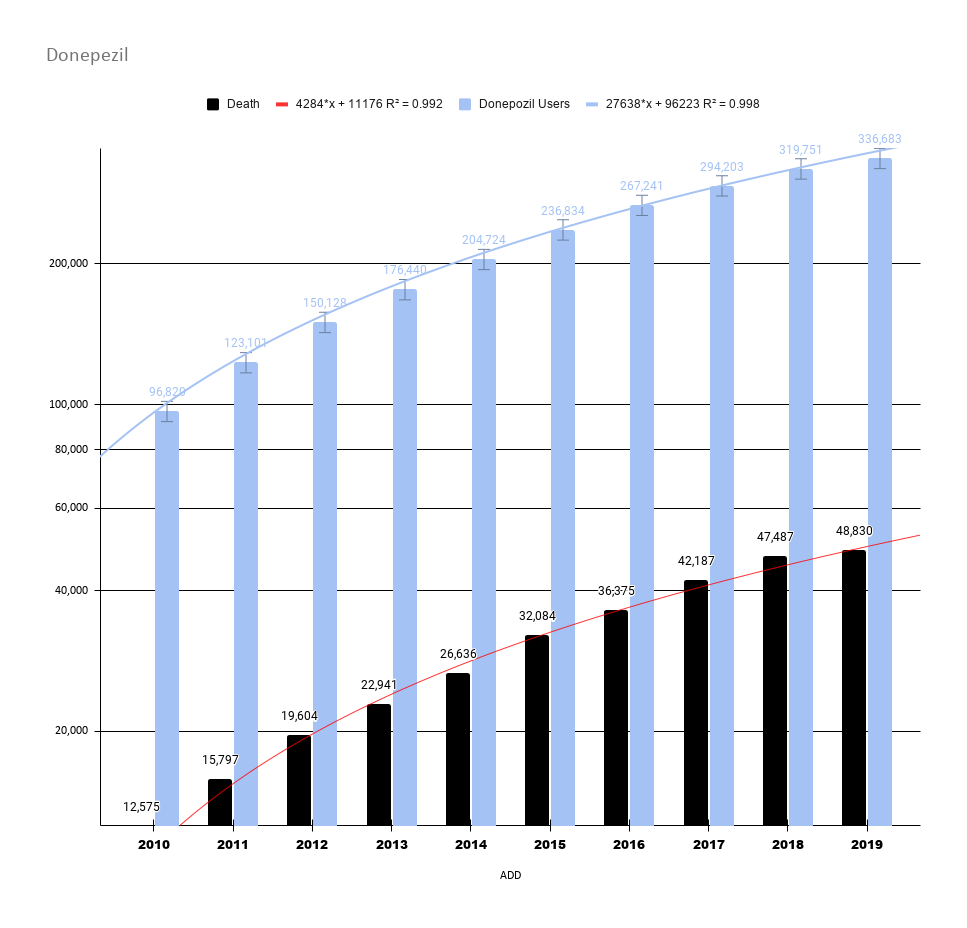
**Fig. S3-1.** Graph of donepezil users and deaths in Korea from 2010 to 2019

The number of users who took donepezil in Korea increased 3.48 times, and the number of deaths increased 3.88 times from 2010 to June 2019. Donepezil users' life expectancies were significantly observed between 2017 and 2019. The DMA was strengthened on June 12 2018.

Table S11. Rivastigmine and User and Death Toll

| year | code | Rivastigmine user | death |
| --- | --- | --- | --- |
| 2010 | 2245 | 8,070 | 780 |
| 2011 | 2245 | 9,788 | 850 |
| 2012 | 2245 | 11,218 | 1,103 |
| 2013 | 2245 | 11,935 | 1,170 |
| 2014 | 2245 | 13,705 | 1,291 |
| 2015 | 2245 | 15,542 | 1,847 |
| 2016 | 2245 | 15,103 | 1,682 |
| 2017 | 2245 | 14,443 | 1,716 |
| 2018 | 2245 | 14,777 | 1,839 |
| 2019 | 2245 | 14,964 | 1,840 |

| year | code | Rivastigmine user | death |
| --- | --- | --- | --- |
| 2010 | 2245 | 100000 | 9665.4 |
| 2011 | 2245 | 100000 | 8684.1 |
| 2012 | 2245 | 100000 | 9832.4 |
| 2013 | 2245 | 100000 | 9803.1 |
| 2014 | 2245 | 100000 | 9419.9 |
| 2015 | 2245 | 100000 | 11883.9 |
| 2016 | 2245 | 100000 | 11136.9 |
| 2017 | 2245 | 100000 | 11881.2 |
| 2018 | 2245 | 100000 | 12445.0 |
| 2019 | 2245 | 100000 | 12296.2 |

(Deaths per 100,000 population)


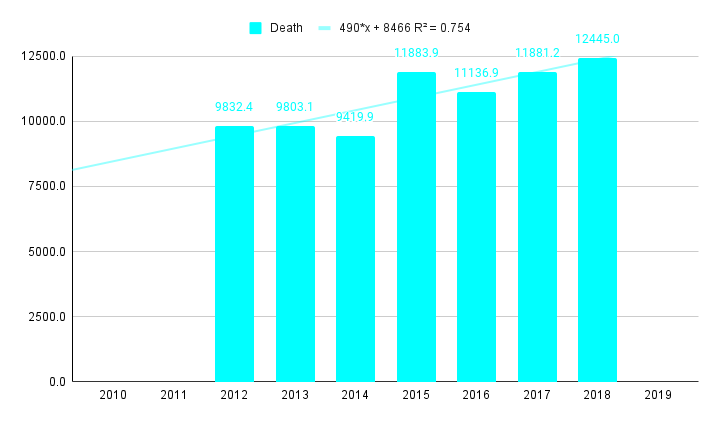

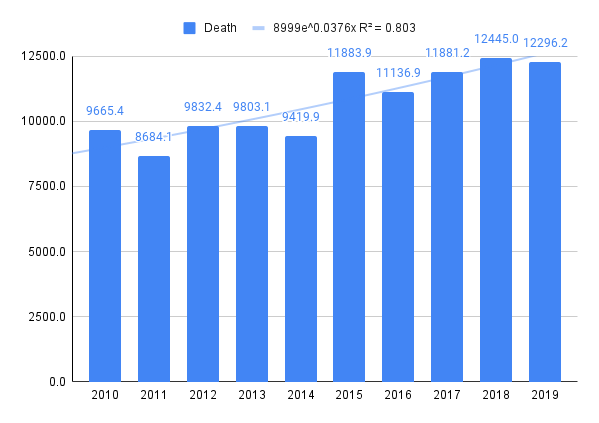


Fig. S4. Rivastigmine Trend line. 3.8E-03*x + 0.133 R^2^ = 0.006


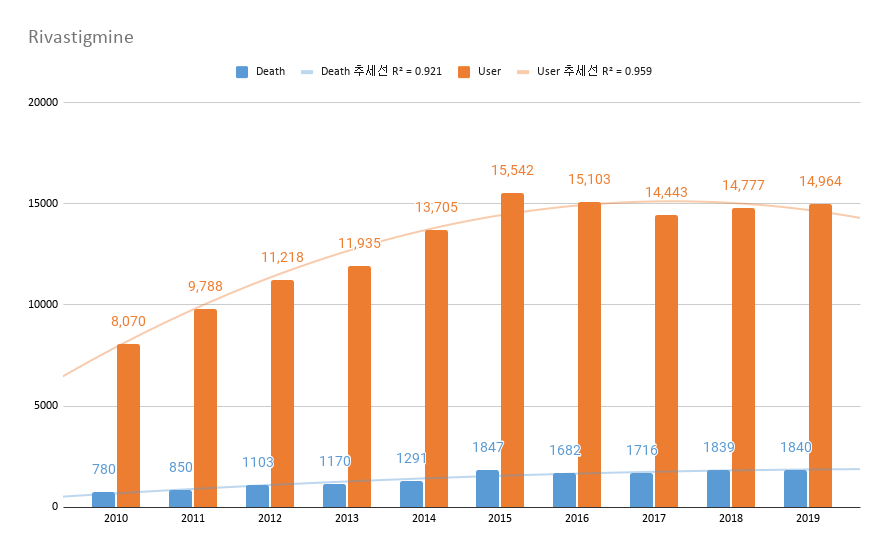


**Fig. S4-1.** Rivastigmine Trend line. The number of users who took rivastigmine in Korea increased 1.84 times, and the number of deaths increased by 2.36 times from 2010 to June 2019

Table S12. Memantine and User and the Death Toll

| year | code | Memantine user | death |
| --- | --- | --- | --- |
| 2010 | 1900 | 31,965 | 5,559 |
| 2011 | 1900 | 35,315 | 5,945 |
| 2012 | 1900 | 37,138 | 6,419 |
| 2013 | 1900 | 38,391 | 6,403 |
| 2014 | 1900 | 43,165 | 6,568 |
| 2015 | 1900 | 52,358 | 8,134 |
| 2016 | 1900 | 58,626 | 9,080 |
| 2017 | 1900 | 65,605 | 10,718 |
| 2018 | 1900 | 70,873 | 11,597 |
| 2019 | 1900 | 79,770 | 12,714 |

| year | code | Memantine user | death |
| --- | --- | --- | --- |
| 2010 | 1900 | 100000 | 17390.9 |
| 2011 | 1900 | 100000 | 16834.2 |
| 2012 | 1900 | 100000 | 17284.2 |
| 2013 | 1900 | 100000 | 16678.4 |
| 2014 | 1900 | 100000 | 15216.0 |
| 2015 | 1900 | 100000 | 15535.4 |
| 2016 | 1900 | 100000 | 15488.0 |
| 2017 | 1900 | 100000 | 16337.2 |
| 2018 | 1900 | 100000 | 16363.1 |
| 2019 | 1900 | 100000 | 15938.3 |

(Deaths per 100,000 population)


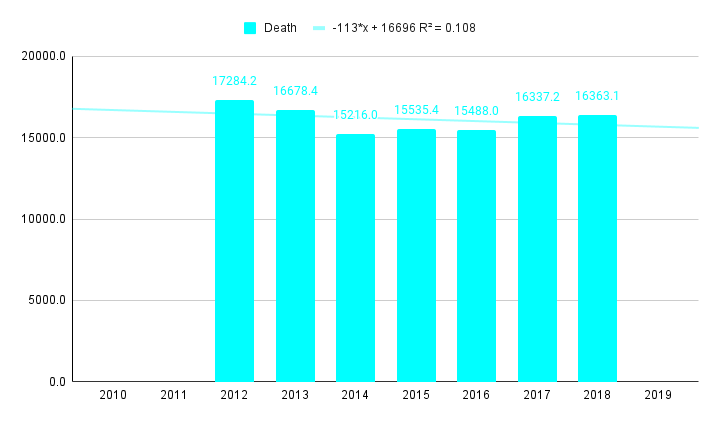

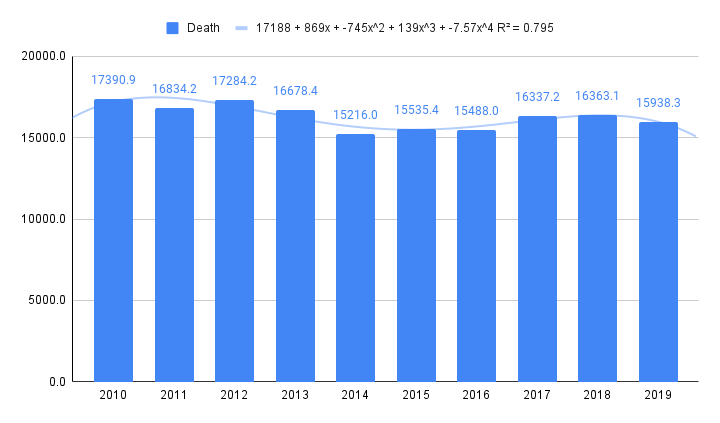


Fig. S5. Memantine Trend line.


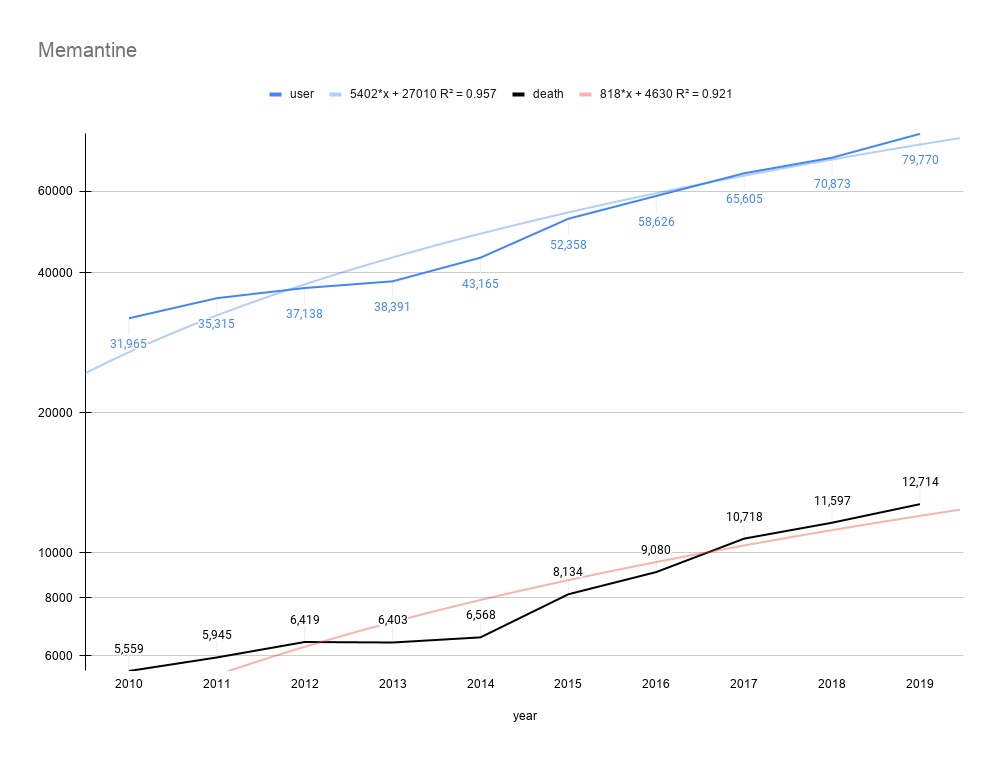


**Fig. S5-1.** Graph with memantine users and deaths in Korea from 2010 to 2019

The number of users who took memantine in Korea increased 2.50 times, and the number of deaths increased by 2.29 times from 2010 to June 2019. Memantine is an uncompetitive NMDA receptor modulator. It is prescribed to treat moderate-to-severe AD.

Table S13. Risperidone and User and Death Toll

| year | code | Risperidone user | death |
| --- | --- | --- | --- |
| 2010 | 2242 | 181,728 | 8,945 |
| 2011 | 2242 | 186,077 | 8,976 |
| 2012 | 2242 | 191,121 | 9,519 |
| 2013 | 2242 | 189,702 | 9,354 |
| 2014 | 2242 | 190,178 | 9,271 |
| 2015 | 2242 | 195,951 | 10,052 |
| 2016 | 2242 | 199,776 | 10,396 |
| 2017 | 2242 | 203,635 | 10,822 |
| 2018 | 2242 | 208,994 | 11,179 |
| 2019 | 2242 | 228,123 | 12,044 |

| year | code | Risperidone user | death |
| --- | --- | --- | --- |
| 2010 | 2242 | 100000 | 4922.2 |
| 2011 | 2242 | 100000 | 4823.8 |
| 2012 | 2242 | 100000 | 4980.6 |
| 2013 | 2242 | 100000 | 4930.9 |
| 2014 | 2242 | 100000 | 4874.9 |
| 2015 | 2242 | 100000 | 5129.9 |
| 2016 | 2242 | 100000 | 5203.8 |
| 2017 | 2242 | 100000 | 5314.4 |
| 2018 | 2242 | 100000 | 5349.0 |
| 2019 | 2242 | 100000 | 5279.6 |

(Deaths per 100,000 population)


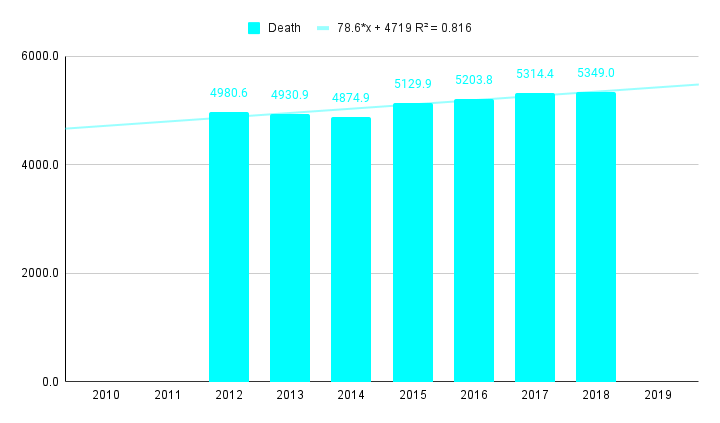

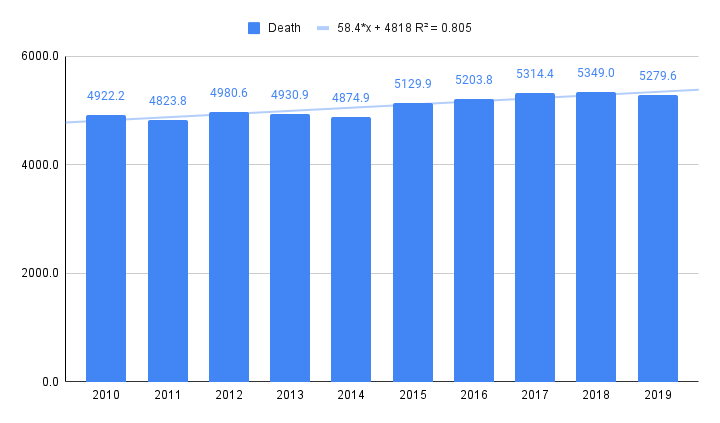


Fig. S6. Risperidone Trend line.


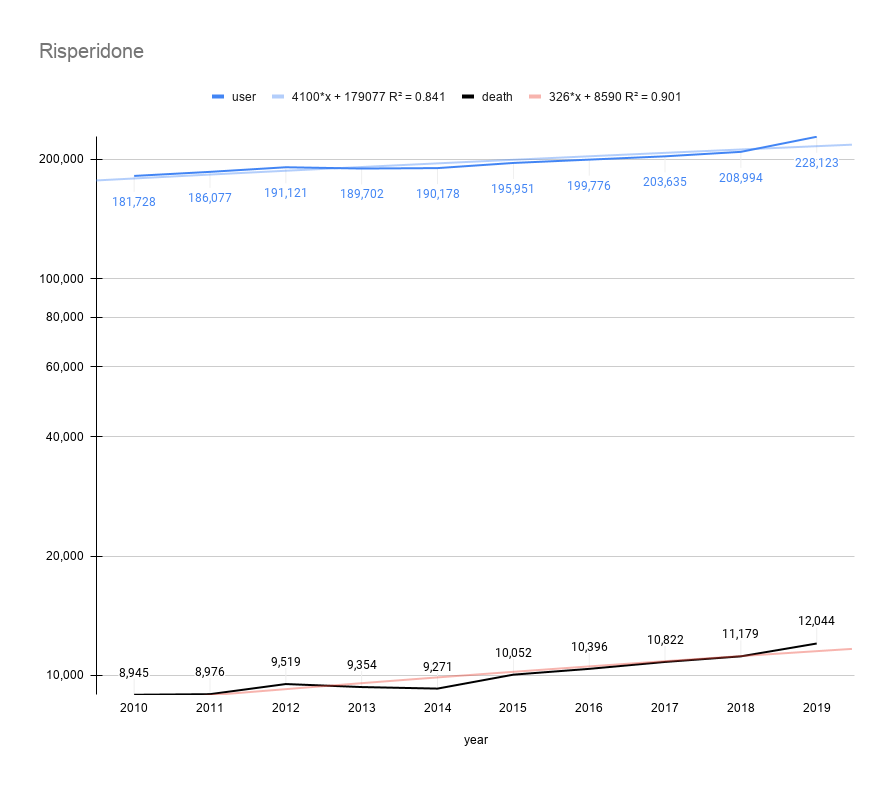


**Fig. S6-1.** Graph with risperidone users and deaths in Korea from 2010 to 2019

The number of users who took risperidone in Korea increased 1.26 times, and the number of deaths increased by 1.35 times from 2010 to June 2019. Risperidone is an antipsychotic medication prescribed to treat schizophrenia and bipolar disorder. It is known to increase the mortality of dementia patients.

Table S14. Fluoxetine and User and Death Toll

| year | code | Fluoxetine user | death |
| --- | --- | --- | --- |
| 2010 | 1802 | 156,899 | 1,802 |
| 2011 | 1802 | 151,252 | 1,580 |
| 2012 | 1802 | 150,654 | 1,582 |
| 2013 | 1802 | 140,194 | 1,439 |
| 2014 | 1802 | 134,013 | 1,395 |
| 2015 | 1802 | 133,262 | 1,373 |
| 2016 | 1802 | 137,846 | 1,329 |
| 2017 | 1802 | 144,496 | 1,268 |
| 2018 | 1802 | 157,170 | 1,369 |
| 2019 | 1802 | 173,284 | 1,405 |

| year | code | Fluoxetine user | death |
| --- | --- | --- | --- |
| 2010 | 1802 | 100000 | 1148.5 |
| 2011 | 1802 | 100000 | 1044.6 |
| 2012 | 1802 | 100000 | 1050.1 |
| 2013 | 1802 | 100000 | 1026.4 |
| 2014 | 1802 | 100000 | 1040.9 |
| 2015 | 1802 | 100000 | 1030.3 |
| 2016 | 1802 | 100000 | 964.1 |
| 2017 | 1802 | 100000 | 877.5 |
| 2018 | 1802 | 100000 | 871.0 |
| 2019 | 1802 | 100000 | 810.8 |

(Deaths per 100,000 population)


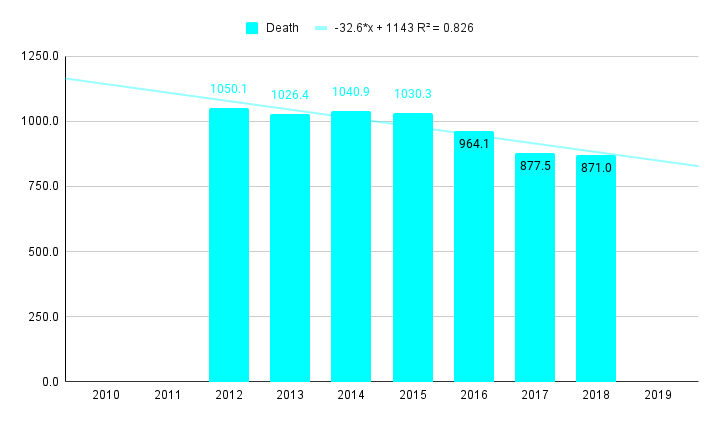

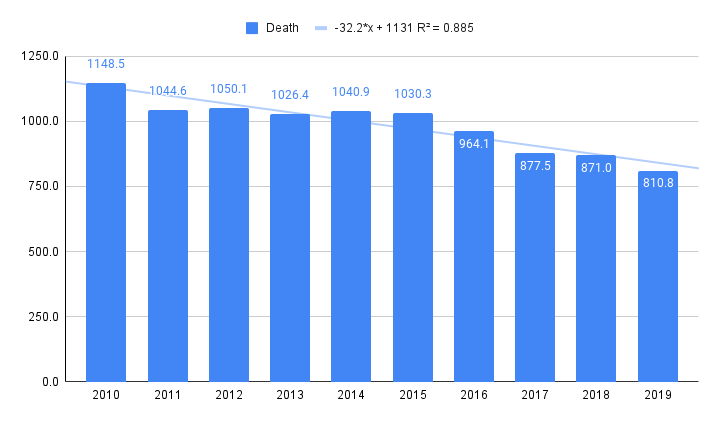


Fig. S7. Fluoxetine Trend line.

Table S15. Olanzapine and User and Death Toll

| year | code | Olanzapine user | death |
| --- | --- | --- | --- |
| 2010 | 2040 | 37,810 | 1,654 |
| 2011 | 2040 | 45,177 | 2,001 |
| 2012 | 2040 | 54,344 | 2,681 |
| 2013 | 2040 | 59,184 | 3,112 |
| 2014 | 2040 | 63,444 | 3,276 |
| 2015 | 2040 | 64,123 | 3,036 |
| 2016 | 2040 | 66,902 | 3,368 |
| 2017 | 2040 | 75,153 | 3,700 |
| 2018 | 2040 | 81,868 | 4,042 |
| 2019 | 2040 | 87,565 | 4,359 |

| year | code | Olanzapine user | death |
| --- | --- | --- | --- |
| 2010 | 2040 | 100000 | 4374.5 |
| 2011 | 2040 | 100000 | 4429.2 |
| 2012 | 2040 | 100000 | 4933.4 |
| 2013 | 2040 | 100000 | 5258.2 |
| 2014 | 2040 | 100000 | 5163.6 |
| 2015 | 2040 | 100000 | 4734.7 |
| 2016 | 2040 | 100000 | 5034.2 |
| 2017 | 2040 | 100000 | 4923.3 |
| 2018 | 2040 | 100000 | 4937.2 |
| 2019 | 2040 | 100000 | 4978.0 |

(Deaths per 100,000 population)


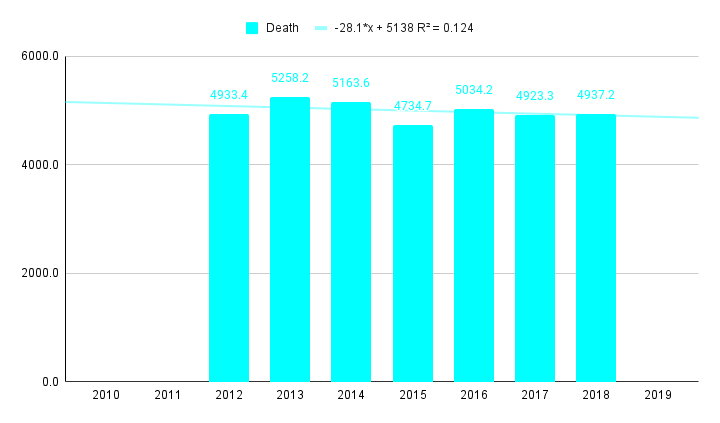

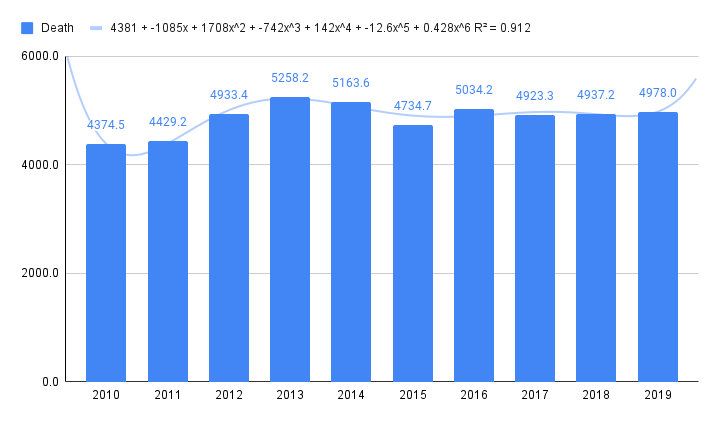


Fig. S8. Olanzapine Trend line.

Table S16. Sertaline and User and Death Toll

| year | code | Sertaline user | death |
| --- | --- | --- | --- |
| 2010 | 2270 | 71,494 | 1,341 |
| 2011 | 2270 | 76,809 | 1,319 |
| 2012 | 2270 | 85,193 | 1,377 |
| 2013 | 2270 | 86,931 | 1,427 |
| 2014 | 2270 | 89,114 | 1,565 |
| 2015 | 2270 | 92,676 | 1,522 |
| 2016 | 2270 | 103,099 | 1,684 |
| 2017 | 2270 | 115,370 | 1,853 |
| 2018 | 2270 | 129,710 | 1,982 |
| 2019 | 2270 | 142,660 | 2,005 |

| year | code | Sertaline user | death |
| --- | --- | --- | --- |
| 2010 | 2270 | 100000 | 1875.7 |
| 2011 | 2270 | 100000 | 1717.2 |
| 2012 | 2270 | 100000 | 1616.3 |
| 2013 | 2270 | 100000 | 1641.5 |
| 2014 | 2270 | 100000 | 1756.2 |
| 2015 | 2270 | 100000 | 1642.3 |
| 2016 | 2270 | 100000 | 1633.4 |
| 2017 | 2270 | 100000 | 1606.1 |
| 2018 | 2270 | 100000 | 1528.0 |
| 2019 | 2270 | 100000 | 1405.4 |

(Deaths per 100,000 population)


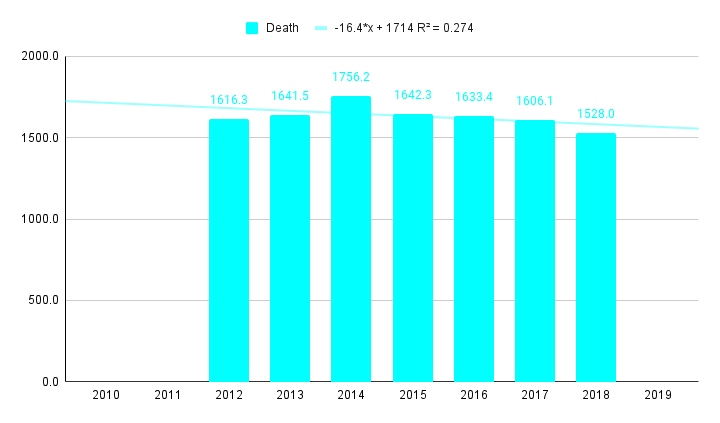

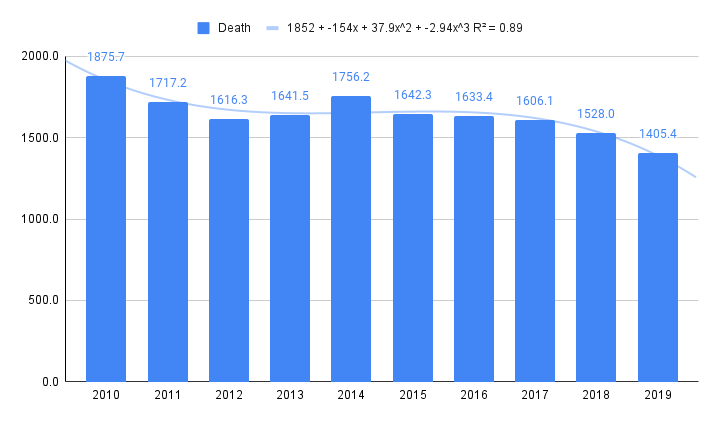


Fig. S9. Sertaline Trend line.

Table S17. Quetiapine and User and Death Toll

| year | code | Quetiapine user | death |
| --- | --- | --- | --- |
| 2010 | 3786 | 140,218 | 10,654 |
| 2011 | 3786 | 172,218 | 13,466 |
| 2012 | 3786 | 202,486 | 17,654 |
| 2013 | 3786 | 228,140 | 21,117 |
| 2014 | 3786 | 259,635 | 24,520 |
| 2015 | 3786 | 290,105 | 27,972 |
| 2016 | 3786 | 331,811 | 32,972 |
| 2017 | 3786 | 384,209 | 39,984 |
| 2018 | 3786 | 439,704 | 46,257 |
| 2019 | 3786 | 540,397 | 51,767 |

| year | code | Quetiapine user | death |
| --- | --- | --- | --- |
| 2010 | 3786 | 100000 | 7598.2 |
| 2011 | 3786 | 100000 | 7819.2 |
| 2012 | 3786 | 100000 | 8718.6 |
| 2013 | 3786 | 100000 | 9256.2 |
| 2014 | 3786 | 100000 | 9444.0 |
| 2015 | 3786 | 100000 | 9642.0 |
| 2016 | 3786 | 100000 | 9937.0 |
| 2017 | 3786 | 100000 | 10406.8 |
| 2018 | 3786 | 100000 | 10520.0 |
| 2019 | 3786 | 100000 | 9579.4 |

(Deaths per 100,000 population)


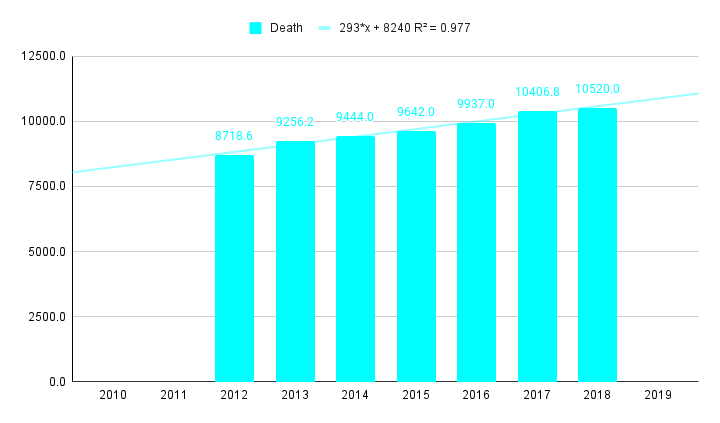

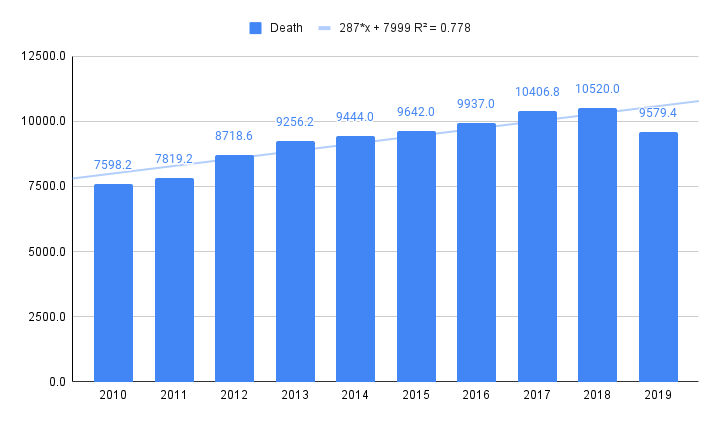


Fig. S10. Quetiapine Trend line.

Table S18. Aripiprazone and User and Death Toll

| year | code | Ariprprazone | death |
| --- | --- | --- | --- |
| 2010 | 4515 | 25,155 | 330 |
| 2011 | 4515 | 28,561 | 382 |
| 2012 | 4515 | 37,290 | 466 |
| 2013 | 4515 | 45,297 | 570 |
| 2014 | 4515 | 67,152 | 768 |
| 2015 | 4515 | 84,102 | 1,086 |
| 2016 | 4515 | 102,241 | 1,164 |
| 2017 | 4515 | 133,075 | 1,448 |
| 2018 | 4515 | 174,861 | 1,895 |
| 2019 | 4515 | 214,761 | 2,211 |

| year | code | Ariprprazone | death |
| --- | --- | --- | --- |
| 2010 | 4515 | 100000 | 1311.9 |
| 2011 | 4515 | 100000 | 1337.5 |
| 2012 | 4515 | 100000 | 1249.7 |
| 2013 | 4515 | 100000 | 1258.4 |
| 2014 | 4515 | 100000 | 1143.7 |
| 2015 | 4515 | 100000 | 1291.3 |
| 2016 | 4515 | 100000 | 1138.5 |
| 2017 | 4515 | 100000 | 1088.1 |
| 2018 | 4515 | 100000 | 1083.7 |
| 2019 | 4515 | 100000 | 1029.5 |

(Deaths per 100,000 population)


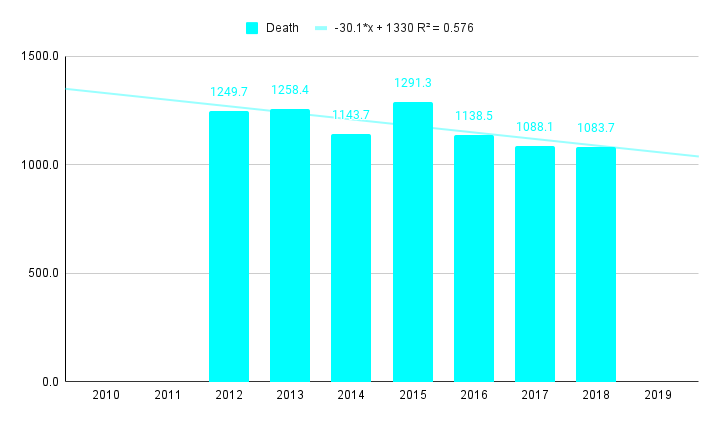

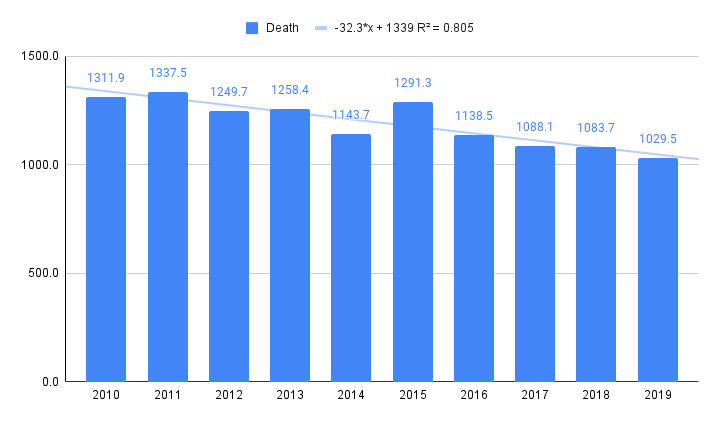


Fig. S11. Aripiprazone Trend line.

Table S19. Escitalopram and User and the Death Toll

| year | code | Escitalopram | death |
| --- | --- | --- | --- |
| 2010 | 4748 | 227,186 | 5,093 |
| 2011 | 4748 | 258,981 | 5,949 |
| 2012 | 4748 | 303,698 | 7,060 |
| 2013 | 4748 | 326,645 | 7,585 |
| 2014 | 4748 | 359,582 | 8,118 |
| 2015 | 4748 | 391,772 | 8,726 |
| 2016 | 4748 | 431,465 | 9,298 |
| 2017 | 4748 | 478,751 | 10,365 |
| 2018 | 4748 | 549,235 | 11,480 |
| 2019 | 4748 | 612,579 | 12,472 |

| year | code | Escitalopram | death |
| --- | --- | --- | --- |
| 2010 | 4748 | 100000 | 2241.8 |
| 2011 | 4748 | 100000 | 2297.1 |
| 2012 | 4748 | 100000 | 2324.7 |
| 2013 | 4748 | 100000 | 2322.1 |
| 2014 | 4748 | 100000 | 2257.6 |
| 2015 | 4748 | 100000 | 2227.3 |
| 2016 | 4748 | 100000 | 2155.0 |
| 2017 | 4748 | 100000 | 2165.0 |
| 2018 | 4748 | 100000 | 2090.2 |
| 2019 | 4748 | 100000 | 2036.0 |

(Deaths per 100,000 population)


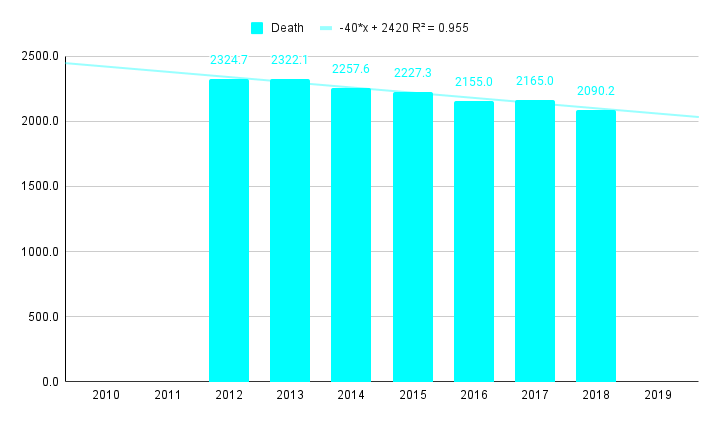

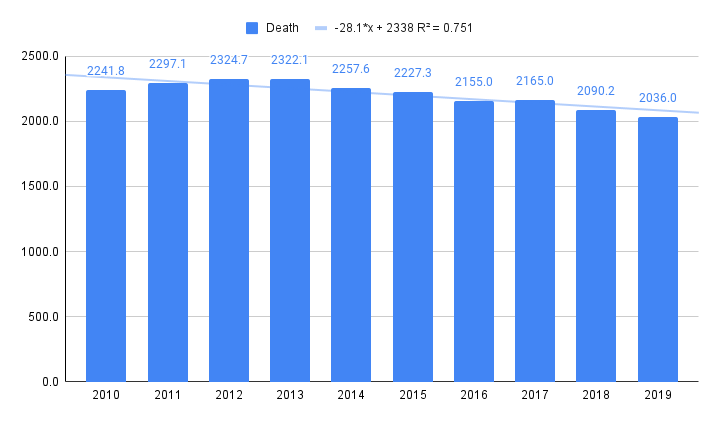


Fig. S12. Escitalopram Trend line.

**S**

Table S20. The others and User and the Death Toll

| year | code | The others user | death |
| --- | --- | --- | --- |
| 2010 | 9999 | 518,890 | 29,795 |
| 2011 | 9999 | 537,684 | 30,788 |
| 2012 | 9999 | 570,704 | 33,246 |
| 2013 | 9999 | 576,421 | 34,654 |
| 2014 | 9999 | 603,698 | 36,134 |
| 2015 | 9999 | 634,889 | 39,692 |
| 2016 | 9999 | 659,428 | 41,003 |
| 2017 | 9999 | 689,476 | 43,792 |
| 2018 | 9999 | 733,711 | 45,972 |
| 2019 | 9999 | 803,750 | 47,585 |

| year | code | The others user | death |
| --- | --- | --- | --- |
| 2010 | 9999 | 100000 | 5742.1 |
| 2011 | 9999 | 100000 | 5726.0 |
| 2012 | 9999 | 100000 | 5825.4 |
| 2013 | 9999 | 100000 | 6011.9 |
| 2014 | 9999 | 100000 | 5985.4 |
| 2015 | 9999 | 100000 | 6251.8 |
| 2016 | 9999 | 100000 | 6218.0 |
| 2017 | 9999 | 100000 | 6351.5 |
| 2018 | 9999 | 100000 | 6265.7 |
| 2019 | 9999 | 100000 | 5920.4 |

(Deaths per 100,000 population)


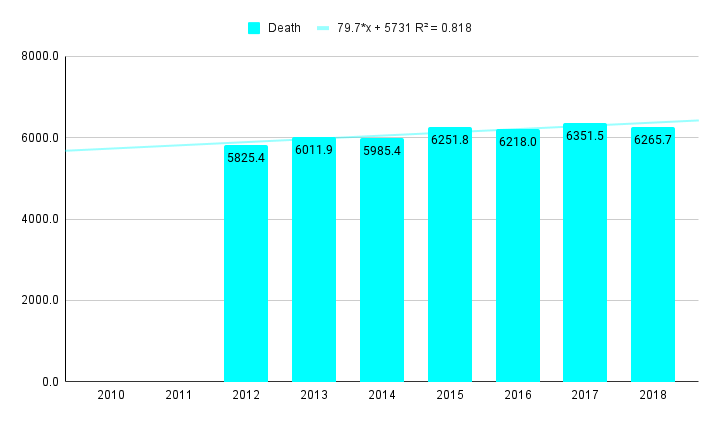

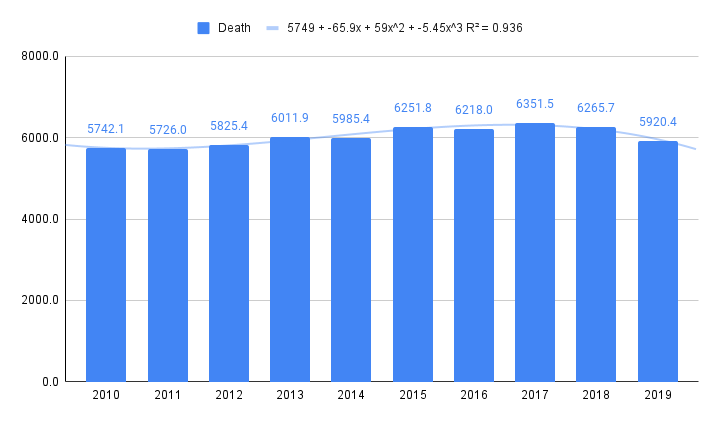


Fig. S13. AAD The others line.


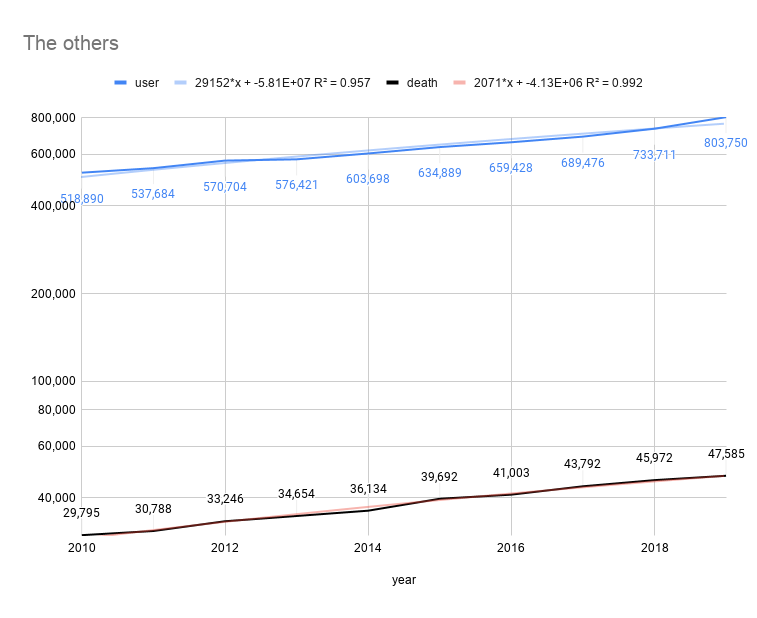


**Fig. S12-1.** Graph with the other users (including galantamine) and deaths in Korea from 2010 to 2019

The number of users who took galantamine and other psychotropic medications in Korea increased 1.55 times, and the number of deaths increased by 1.60 times from 2010 to June 2019.

# Supplement Section 4. Statistics

Table S21. NHIS Dementia Medicines: Users and Deaths Toll

| Year | | AAD group 1 | | | | Donepezil | | | | Rivastigmine | | | | Memantine | | | |  |
| --- | --- | --- | --- | --- | --- | --- | --- | --- | --- | --- | --- | --- | --- | --- | --- | --- | --- | --- |
|  | User | | Death | User | | | Death | | User | | Death | | user | | | death | |  |
| 2010 | 136,855 | | 18,914 | 96,820 | | | 12,575 | | 8,070 | | 780 | | 31,965 | | | 5,559 | |  |
| 2011 | 168,204 | | 22,592 | 123,101 | | | 15,797 | | 9,788 | | 850 | | 35,315 | | | 5,945 | |  |
| 2012 | 198,484 | | 27,126 | 150,128 | | | 19,604 | | 11,218 | | 1,103 | | 37,138 | | | 6,419 | |  |
| 2013 | 226,766 | | 30,514 | 176,440 | | | 22,941 | | 11,935 | | 1,170 | | 38,391 | | | 6,403 | |  |
| 2014 | 261,594 | | 34,495 | 204,724 | | | 26,636 | | 13,705 | | 1,291 | | 43,165 | | | 6,568 | |  |
| 2015 | 304,734 | | 42,065 | 236,834 | | | 32,084 | | 15,542 | | 1,847 | | 52,358 | | | 8,134 | |  |
| 2016 | 340,970 | | 47,137 | 267,241 | | | 36,375 | | 15,103 | | 1,682 | | 58,626 | | | 9,080 | |  |
| 2017 | 374,251 | | 54,621 | 294,203 | | | 42,187 | | 14,443 | | 1,716 | | 65,605 | | | 10,718 | |  |
| 2018 | 405,401 | | 60,923 | 319,751 | | | 47,487 | | 14,777 | | 1,839 | | 70,873 | | | 11,597 | |  |
| 2019 | 431,417 | | 63,384 | 336,683 | | | 48,830 | | 14,964 | | 1,840 | | 79,770 | | | 12,714 | |  |
|  | | | | | | | | Results of mortality | | | | | | | | | |  |
| Year | | AAD group 1 | | | | Donepezil | | | | Rivastigmine | | | | Memantine | | | |  |
|  | User | | Death | User | | | Death | | User | | Death | | user | | | death | |  |
| 2010 |  | | 0.1382 |  | | | 0.1299 | |  | | 0.0967 | |  | | | 0.1739 | |  |
| 2011 |  | | 0.1343 |  | | | 0.1283 | |  | | 0.0868 | |  | | | 0.1683 | |  |
| 2012 |  | | 0.1367 |  | | | 0.1306 | |  | | 0.0983 | |  | | | 0.1728 | |  |
| 2013 |  | | 0.1346 |  | | | 0.1300 | |  | | 0.0980 | |  | | | 0.1668 | |  |
| 2014 |  | | 0.1319 |  | | | 0.1301 | |  | | 0.0942 | |  | | | 0.1522 | |  |
| 2015 |  | | 0.1380 |  | | | 0.1355 | |  | | 0.1188 | |  | | | 0.1554 | |  |
| 2016 |  | | 0.1382 |  | | | 0.1361 | |  | | 0.1114 | |  | | | 0.1549 | |  |
| 2017 |  | | 0.1459 |  | | | 0.1434 | |  | | 0.1188 | |  | | | 0.1634 | |  |
| 2018 |  | | 0.1503 |  | | | 0.1485 | |  | | 0.1245 | |  | | | 0.1636 | |  |
| 2019 |  | | 0.1469 |  | | | 0.1450 | |  | | 0.1230 | |  | | | 0.1594 | |  |
| **Summary of Data** | | | | | | | | | | | | | | | | | | |
|  | | | ***Treatments*** | | | | | | | | | | | | | | | |
|  |  |  | 1 | | 2 | | | | 3 | | | 4 | | | 5 | | Total | |
| N | | | 10 | | 10 | | | | 10 | | | 10 | | |  | | 40 | |
| ∑X | | | 1.395 | | 1.3574 | | | | 1.0705 | | | 1.6307 | | |  | | 5.4536 | |
| Mean | | | 0.1395 | | 0.1357 | | | | 0.1071 | | | 0.1631 | | |  | | 0.136 | |
| ∑X^2^ | | | 0.1949 | | 0.1847 | | | | 0.1163 | | | 0.2664 | | |  | | 0.7624 | |
| Std.Dev. | | | 0.0061 | | 0.0074 | | | | 0.0137 | | | 0.0075 | | |  | | 0.022 | |
| **Result Details** | | | | | | | | | | | | | | | | | | |
| ***Source*** | | | | | ***SS*** | | | | ***df*** | | | ***MS*** | | |  | | | |
| Between-treatments | | | | | 0.0158 | | | | 3 | | | 0.0053 | | | *F* = 62.7191 | | | |
| Within-treatments | | | | | 0.003 | | | | 36 | | | 0.0001 | | |  | | | |
| Total | | | | | 0.0189 | | | | 39 | | |  | | |  | | | |

The *f*-ratio value is 62.7191. The *p*-value is < .00001. The result is significant at *p* < .05.

The values of the Friedman X^2^_r_ statistic and p appear


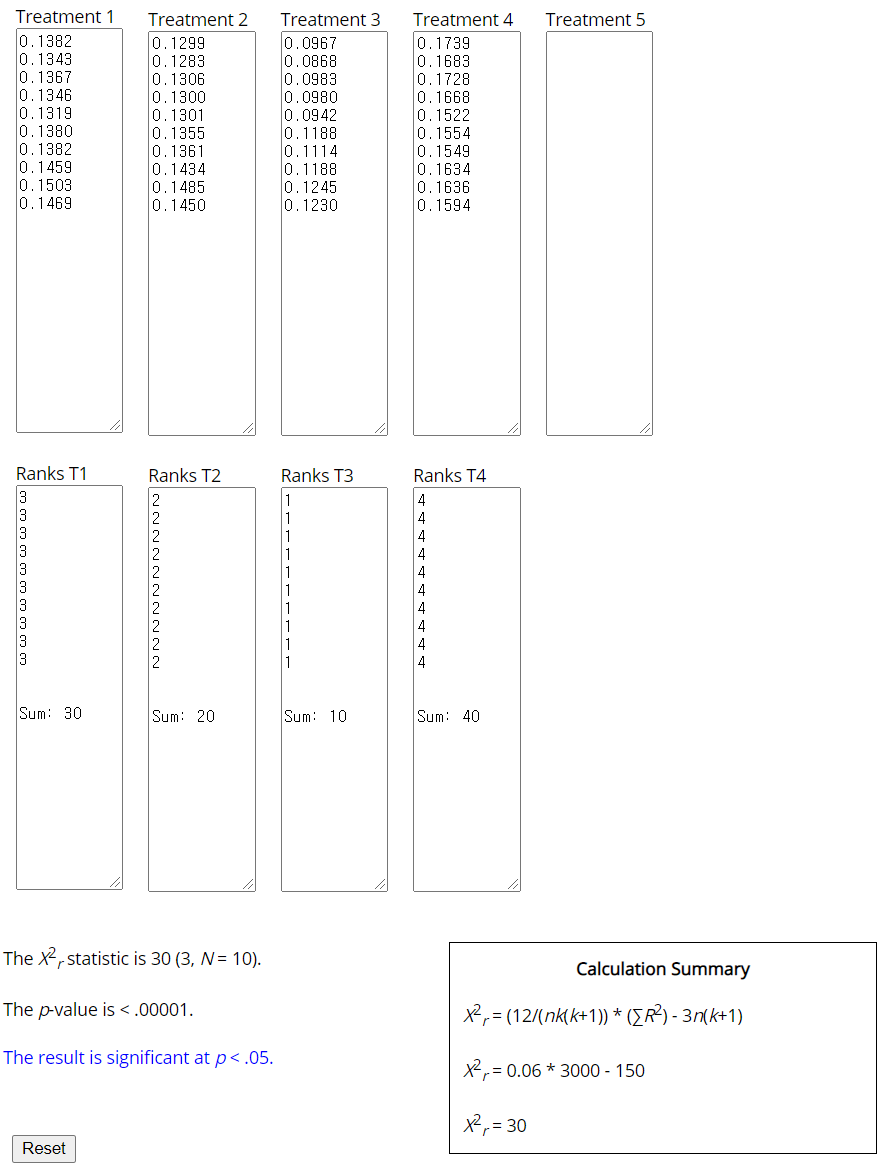


Table S19-1. NHIS Dementia Medicines: Users and Deaths Toll

| Year | AAD | | Fluoxetine | | Olanzapine | | Sertraline | | Quetiapine | | Aripiprazole | | Escitalopram | | The others | |
| --- | --- | --- | --- | --- | --- | --- | --- | --- | --- | --- | --- | --- | --- | --- | --- | --- |
|  | User | Death | user | death | user | death | user | death | user | death | user | death | user | death | user | death |
| 2010 | 1,496,235 | 78,528 | 156,899 | 1,802 | 37,810 | 1,654 | 71,494 | 1,341 | 140,218 | 10,654 | 25,155 | 330 | 227,186 | 5,093 | 518,890 | 29,795 |
| 2011 | 1,624,963 | 87,053 | 151,252 | 1,580 | 45,177 | 2,001 | 76,809 | 1,319 | 172,218 | 13,466 | 28,561 | 382 | 258,981 | 5,949 | 537,684 | 30,788 |
| 2012 | 1,793,974 | 100,711 | 150,654 | 1,582 | 54,344 | 2,681 | 85,193 | 1,377 | 202,486 | 17,654 | 37,290 | 466 | 303,698 | 7,060 | 570,704 | 33,246 |
| 2013 | 1,879,280 | 109,772 | 140,194 | 1,439 | 59,184 | 3,112 | 86,931 | 1,427 | 228,140 | 21,117 | 45,297 | 570 | 326,645 | 7,585 | 576,421 | 34,654 |
| 2014 | 2,028,410 | 119,542 | 134,013 | 1,395 | 63,444 | 3,276 | 89,114 | 1,565 | 259,635 | 24,520 | 67,152 | 768 | 359,582 | 8,118 | 603,698 | 36,134 |
| 2015 | 2,191,614 | 135,524 | 133,262 | 1,373 | 64,123 | 3,036 | 92,676 | 1,522 | 290,105 | 27,972 | 84,102 | 1,086 | 391,772 | 8,726 | 634,889 | 39,692 |
| 2016 | 2,373,538 | 148,351 | 137,846 | 1,329 | 66,902 | 3,368 | 103,099 | 1,684 | 331,811 | 32,972 | 102,241 | 1,164 | 431,465 | 9,298 | 659,428 | 41,003 |
| 2017 | 2,598,416 | 167,853 | 144,496 | 1,268 | 75,153 | 3,700 | 115,370 | 1,853 | 384,209 | 39,984 | 133,075 | 1,448 | 478,751 | 10,365 | 689,476 | 43,792 |
| 2018 | 2,880,654 | 185,099 | 157,170 | 1,369 | 81,868 | 4,042 | 129,710 | 1,982 | 439,704 | 46,257 | 174,861 | 1,895 | 549,235 | 11,480 | 733,711 | 45,972 |
| 2019 | 3,234,536 | 197,232 | 173,284 | 1,405 | 87,565 | 4,359 | 142,660 | 2,005 | 540,397 | 51,767 | 214,761 | 2,211 | 612,579 | 12,472 | 803,750 | 47,585 |

Table S22. NHIS Dementia Medicines: Users and Deaths Toll

| Year | AAD | | Donepezil | | Rivastigmine | | Memantine | | Risperidone | |
| --- | --- | --- | --- | --- | --- | --- | --- | --- | --- | --- |
|  | User | Death | User | Death | User | Death | user | death | user | death |
| 2010 | 1,496,235 | 78,528 | 96,820 | 12,575 | 8,070 | 780 | 31,965 | 5,559 | 181728 | 8945 |
| 2011 | 1,624,963 | 87,053 | 123,101 | 15,797 | 9,788 | 850 | 35,315 | 5,945 | 186077 | 8976 |
| 2012 | 1,793,974 | 100,711 | 150,128 | 19,604 | 11,218 | 1,103 | 37,138 | 6,419 | 191121 | 9519 |
| 2013 | 1,879,280 | 109,772 | 176,440 | 22,941 | 11,935 | 1,170 | 38,391 | 6,403 | 189702 | 9354 |
| 2014 | 2,028,410 | 119,542 | 204,724 | 26,636 | 13,705 | 1,291 | 43,165 | 6,568 | 190178 | 9271 |
| 2015 | 2,191,614 | 135,524 | 236,834 | 32,084 | 15,542 | 1,847 | 52,358 | 8,134 | 195951 | 10052 |
| 2016 | 2,373,538 | 148,351 | 267,241 | 36,375 | 15,103 | 1,682 | 58,626 | 9,080 | 199776 | 10396 |
| 2017 | 2,598,416 | 167,853 | 294,203 | 42,187 | 14,443 | 1,716 | 65,605 | 10,718 | 203635 | 10822 |
| 2018 | 2,880,654 | 185,099 | 319,751 | 47,487 | 14,777 | 1,839 | 70,873 | 11,597 | 208994 | 11179 |
| 2019 | 3,234,536 | 197,232 | 336,683 | 48,830 | 14,964 | 1,840 | 79,770 | 12,714 | 228123 | 12044 |

# Supplement Section 5. Dementia Demography of NHIS from Central Dementia Center.

| Number of people with dementia | | | | | | | | |  |
| --- | --- | --- | --- | --- | --- | --- | --- | --- | --- |
| ▶ Source: National Health Insurance Corporation (2019) ▶ Extraction criteria: In 2019, NHIS extracted Dementia care: Inpatient, outpatient, and drug prescription with dementia disease code assigned as main disease code based on ICD codes: F00, F01, F02, F03 , G30 and G31. | | | | | | | | |  |
|  |  |  |  |  |  |  |  | ( Unit: person) |  |
| division | | all | gender | | age | | | |  |
|  |  |  | male | female | under 60 | 60+ | under 65 | 65 years or older |  |
| Nationwide | | 880,371 | 253,813 | 626,558 | 44,576 | 835,795 | 94,187 | 786,184 |  |
| Seoul | | 127,676 | 38,732 | 88,944 | 7,866 | 119,810 | 16,848 | 110,828 |  |
| Busan | | 63,187 | 18,410 | 44,777 | 3,258 | 59,929 | 7,086 | 56,101 |  |
| Daegu Metropolitan City | | 40,093 | 11,192 | 28,901 | 2,340 | 37,753 | 4,857 | 35,236 |  |
| Incheon Metropolitan City | | 40,350 | 11,486 | 28,864 | 2,780 | 37,570 | 5,618 | 34,732 |  |
| Gwangju | | 24,157 | 6,963 | 17,194 | 1,222 | 22,935 | 2,629 | 21,528 |  |
| Daejeon | | 22,160 | 6,504 | 15,656 | 1,110 | 21,050 | 2,361 | 19,799 |  |
| Ulsan Metropolitan City | | 13,765 | 3,805 | 9,960 | 844 | 12,921 | 1,755 | 12,010 |  |
| Sejong Special Self-Governing City | | 3,829 | 1,112 | 2,717 | 170 | 3,659 | 338 | 3,491 |  |
| Gyeonggi-do | | 171,027 | 50,773 | 120,254 | 10,059 | 160,968 | 20,525 | 150,502 |  |
| Gangwon-do | | 30,369 | 8,741 | 21,628 | 1,404 | 28,965 | 3,121 | 27,248 |  |
| Chung-cheong bukdo | | 33,970 | 10,007 | 23,963 | 1,926 | 32,044 | 3,904 | 30,066 |  |
| Chungcheongnam-do | | 49,678 | 14,858 | 34,820 | 1,798 | 47,880 | 3,900 | 45,778 |  |
| Jeollabuk do | | 50,338 | 14,292 | 36,046 | 1,654 | 48,684 | 3,541 | 46,797 |  |
| Jeollanam-do | | 64,560 | 17,682 | 46,878 | 2,420 | 62,140 | 5,233 | 59,327 |  |
| Gyeongsangbuk-do | | 64,410 | 17,835 | 46,575 | 2,138 | 62,272 | 4,871 | 59,539 |  |
| Gyeongsangnam-do | | 70,201 | 18,612 | 51,589 | 3,148 | 67,053 | 6,688 | 63,513 |  |
| Jeju Special Self-Governing Province | | 10,601 | 2,809 | 7,792 | 439 | 10,162 | 912 | 9,689 |  |

( Unit: person )

▶ Source : National Health Insurance Corporation (2010-2019)

▶ Extraction criteria :

In 2019, NHIS extracted Dementia care: Inpatient, outpatient, and drug prescription with dementia disease code assigned as main disease code based on ICD codes: F00, F01, F02, F03 , G30 and G31. The status of the total number of persons with dementia who are eligible for health insurance and those who are eligible for medical benefits, changes in qualifications are not reflected.

( Unit: person )

|  |  | 2010 | 2011 | 2012 | 2013 | 2014 | 2015 | 2016 | 2017 | 2018 | 2019 |
| --- | --- | --- | --- | --- | --- | --- | --- | --- | --- | --- | --- |
| gender | age group |  |  |  |  |  |  |  |  |  |  |
|  | all | 285,773 | 341,889 | 395,901 | 450,917 | 505,555 | 577,427 | 664,475 | 755,871 | 859,132 | 959,001 |
|  | under 60 | 13,388 | 15,179 | 17,718 | 20,293 | 21,434 | 24,114 | 30,168 | 37,453 | 40,624 | 42,812 |
|  | 60+ | 272,385 | 326,710 | 378,183 | 430,624 | 484,121 | 553,313 | 634,307 | 718,418 | 818,508 | 916,189 |
|  | under 65 | 26,426 | 29,668 | 33,751 | 38,081 | 40,583 | 46,925 | 59,446 | 73,349 | 83,950 | 94,196 |
|  | 65 or older | 259,347 | 312,221 | 362,150 | 412,836 | 464,972 | 530,502 | 605,029 | 682,522 | 775,182 | 864,805 |
| total | 40-44 | 871 | 845 | 975 | 1,081 | 1,003 | 1,032 | 1,243 | 1,340 | 1,410 | 1,345 |
|  | 45-49 | 1,778 | 1,854 | 2,050 | 2,366 | 2,247 | 2,487 | 3,059 | 3,679 | 3,784 | 3,752 |
|  | 50-54 | 4,179 | 4,569 | 5,360 | 5,930 | 6,026 | 6,493 | 7,895 | 9,577 | 10,198 | 11,045 |
|  | 55-59 | 6,560 | 7,911 | 9,333 | 10,916 | 12,158 | 14,102 | 17,971 | 22,857 | 25,232 | 26,670 |
|  | 60-64 | 13,038 | 14,489 | 16,033 | 17,788 | 19,149 | 22,811 | 29,278 | 35,896 | 43,326 | 51,384 |
|  | 65-69 | 25,822 | 28,477 | 29,317 | 32,019 | 35,334 | 41,401 | 49,050 | 55,789 | 65,183 | 75,238 |
|  | 70-74 | 46,545 | 54,193 | 62,462 | 68,110 | 71,641 | 78,055 | 84,228 | 87,329 | 97,844 | 110,037 |
|  | 75-79 | 63,159 | 76,221 | 87,800 | 100,172 | 111,144 | 122,742 | 137,466 | 155,853 | 172,229 | 183,020 |
|  | 80-84 | 61,583 | 75,299 | 88,301 | 100,770 | 115,608 | 134,774 | 156,337 | 175,339 | 199,795 | 222,812 |
|  | 85-89 | 41,961 | 51,528 | 60,759 | 70,658 | 83,802 | 97,973 | 113,031 | 130,807 | 149,474 | 169,975 |
|  | 90-94 | 15,996 | 20,886 | 26,429 | 32,818 | 37,591 | 43,894 | 50,524 | 59,281 | 68,610 | 79,724 |
|  | 95-99 | 3,713 | 4,871 | 6,108 | 7,154 | 8,539 | 10,096 | 12,457 | 15,782 | 19,445 | 21,027 |
|  | over 100 | 568 | 746 | 974 | 1,135 | 1,313 | 1,567 | 1936 | 2,342 | 2,602 | 2,972 |
| male | 40-44 | 473 | 475 | 516 | 559 | 478 | 513 | 584 | 635 | 655 | 572 |
|  | 45-49 | 969 | 1006 | 1,041 | 1,167 | 1,077 | 1,163 | 1,327 | 1,548 | 1,520 | 1,461 |
|  | 50-54 | 2,088 | 2,294 | 2,415 | 2,554 | 2,582 | 2,726 | 3,204 | 3,621 | 3,631 | 3,861 |
|  | 55-59 | 3,208 | 3,794 | 4,157 | 4,693 | 5,194 | 5,827 | 7,072 | 8,679 | 8,990 | 9,154 |
|  | 60-64 | 6,159 | 6,638 | 7,000 | 7,419 | 7,993 | 9,165 | 11,512 | 14,000 | 16,088 | 18,393 |
|  | 65-69 | 10,261 | 11,382 | 11,671 | 12,707 | 14,169 | 16,530 | 19,372 | 21,617 | 24,344 | 27,509 |
|  | 70-74 | 16,436 | 19,062 | 22,095 | 24,060 | 25,210 | 27,679 | 30,349 | 32,061 | 35,968 | 40,676 |
|  | 75-79 | 19,122 | 23,001 | 26,685 | 30,924 | 34,506 | 38,289 | 43,540 | 50,237 | 55,925 | 60,569 |
|  | 80-84 | 15,417 | 18,401 | 21,836 | 25,107 | 29,175 | 35,154 | 41,675 | 47,402 | 55,285 | 63,085 |
|  | 85-89 | 9,230 | 11,104 | 12,936 | 14,878 | 17,306 | 19,918 | 23,097 | 27,452 | 32,556 | 37,928 |
|  | 90-94 | 3,017 | 3,946 | 4,950 | 6,023 | 6,861 | 7,782 | 8,796 | 10,121 | 11,839 | 13,835 |
|  | 95-99 | 537 | 684 | 867 | 1,024 | 1,272 | 1,576 | 1,939 | 2,438 | 2,968 | 3,141 |
|  | over 100 | 77 | 99 | 124 | 135 | 146 | 178 | 211 | 259 | 313 | 384 |
| female | 40-44 | 398 | 370 | 459 | 522 | 525 | 519 | 659 | 705 | 755 | 773 |
|  | 45-49 | 809 | 848 | 1,009 | 1,199 | 1,170 | 1,324 | 1,732 | 2,131 | 2,264 | 2,291 |
|  | 50-54 | 2,091 | 2,275 | 2,945 | 3,376 | 3,444 | 3,767 | 4,691 | 5,956 | 6,567 | 7,184 |
|  | 55-59 | 3,352 | 4,117 | 5,176 | 6,223 | 6,964 | 8,275 | 10,899 | 14,178 | 16,242 | 17,516 |
|  | 60-64 | 6,879 | 7,851 | 9,033 | 10,369 | 11,156 | 13,646 | 17,766 | 21,896 | 27,238 | 32,991 |
|  | 65-69 | 15,561 | 17,095 | 17,646 | 19,312 | 21,165 | 24,871 | 29,678 | 34,172 | 40,839 | 47,729 |
|  | 70-74 | 30,109 | 35,131 | 40,367 | 44,050 | 46,431 | 50,376 | 53,879 | 55,268 | 61,876 | 69,361 |
|  | 75-79 | 44,037 | 53,220 | 61,115 | 69,248 | 76,638 | 84,453 | 93,926 | 105,616 | 116,304 | 122,451 |
|  | 80-84 | 46,166 | 56,898 | 66,465 | 75,663 | 86,433 | 99,620 | 114,662 | 127,937 | 144,510 | 159,727 |
|  | 85-89 | 32,731 | 40,424 | 47,823 | 55,780 | 66,496 | 78,055 | 89,934 | 103,355 | 116,918 | 132,047 |
|  | 90-94 | 12,979 | 16,940 | 21,479 | 26,795 | 30,730 | 36,112 | 41,728 | 49,160 | 56,771 | 65,889 |
|  | 95-99 | 3,176 | 4,187 | 5,241 | 6,130 | 7,267 | 8,520 | 10,518 | 13,344 | 16,477 | 17,886 |
|  | over 100 | 491 | 647 | 850 | 1,000 | 1,167 | 1,389 | 1,725 | 2,083 | 2,289 | 2,588 |

Current status of long-term care for the elderly with dementia

▶ Source : National Health Insurance Corporation (2019)

▶ Extraction criteria :

In 2019, NHIS extracted Dementia care: Inpatient, outpatient, and drug prescription with dementia disease code assigned as main disease code based on ICD codes: F00, F01, F02, F03 , G30 and G31.

|  |  | gender | Age -specific section | recipients Total | recipients | | | | | |  |
| --- | --- | --- | --- | --- | --- | --- | --- | --- | --- | --- | --- |
|  |  |  |  |  | **classification** | | | | | |  |
|  |  |  |  |  | **Grade 1** | **2nd grade** | **3rd grade** | **4th grade** | **5th grade** | Supportive |  |
|  |  |  |  | **352,165** | **17,630** | **42,269** | **105,418** | **133849** | **48,771** | **4,228** |  |
| Nationwide | | male | 1-59 | 1,483 | 125 | 196 | 527 | 468 | 147 | 20 |  |
|  |  | male | 60-64 | 2,490 | 161 | 334 | 831 | 851 | 288 | 25 |  |
|  |  | male | 65-69 | 4,949 | 269 | 530 | 1,612 | 1,827 | 652 | 59 |  |
|  |  | male | 70-74 | 8,751 | 442 | 953 | 2,781 | 3,282 | 1,196 | 97 |  |
|  |  | male | 75 years or older | 69,112 | 2,460 | 7,044 | 21,088 | 27,585 | 10,066 | 869 |  |
|  |  | female | 1-59 | 1,233 | 167 | 168 | 428 | 315 | 135 | 20 |  |
|  |  | female | 60-64 | 2,097 | 264 | 258 | 665 | 594 | 280 | 36 |  |
|  |  | female | 65-69 | 4,775 | 407 | 555 | 1,315 | 1,641 | 762 | 95 |  |
|  |  | female | 70-74 | 11,929 | 741 | 1,252 | 3,118 | 4,435 | 2,136 | 247 |  |
|  |  | female | 75 years or older | 245,346 | 12,594 | 30,979 | 73,053 | 92,851 | 33,109 | 2,760 |  |

( Unit: person )

Dementia Relief Center Registration Status by Subject

▶ Source : Dementia Relief Center (2019)

▶ Extraction standard

1) As of Statistics (~ '19 .12.31) ANSYS chlorotic advantage of the features and chlorotic one except person (including death) a person with parental

2) Diagnosis undecided: None of the subjects registered in ANSYS on the statistical basis date have ever been diagnosed with dementia or Screening results cognitive decline and early screening after of non - total number of subjects

3) Normal: Among the subjects registered in ANSYS on the statistical basis date, the external diagnosis result is normal or Total number of subjects who were normal as a result of early examination by the Dementia Relief Center

4) Mild cognitive impairment: As a result of external diagnosis among subjects registered in ANSYS on the statistical basis date, mild cognitive impairment or Dementia relief centers perform early screening results of the sum of mild cognitive impaired subjects

5) Dementia (external diagnosis ): The total number of subjects who were diagnosed with dementia at an external hospital other than the early diagnosis of the dementia safety center among subjects registered in ANSYS on the statistical basis date

6) Dementia (Safety Center Diagnosis ): The total number of subjects registered in ANSYS on the statistical basis date for dementia diagnosis as a result of the 2nd stage of early diagnosis diagnostic test or differential test performed by the Dementia Safety Center

7) Guardians: Total number of guardians registered in ANSYS on the statistical basis date

( Unit: person )

| division | Total number of registrants | subject | | | | | | | guardian |
| --- | --- | --- | --- | --- | --- | --- | --- | --- | --- |
|  |  | **Total subjects** | **Diagnosis undecided** | **normal** | **mild cognitive impairment** | **dementia patient** | | |  |
|  |  |  |  |  |  | **Subtotal** | **external diagnosis** | **Safety Center Diagnosis** |  |
| Nationwide | **3,459,423** | **3,360,816** | **165,138** | **2,663,512** | **111,154** | **421,012** | **276,814** | **144,198** | **98,607** |
| Seoul | **832,374** | 824,338 | 46,606 | 665,989 | 53,438 | 58,305 | 14,042 | 44,263 | 8,036 |
| Busan | **190,919** | 182,486 | 7,875 | 148,067 | 5,718 | 20,826 | 13,914 | 6,912 | 8,433 |
| Daegu Metropolitan City | **178,799** | 177,030 | 12,815 | 143,570 | 3,251 | 17,394 | 11,196 | 6198 | 1,769 |
| Incheon Metropolitan City | **111,560** | 106,997 | 6,616 | 82,603 | 2,188 | 15,590 | 12,225 | 3,365 | 4,563 |
| Gwangju | **62,580** | 61,298 | 3,156 | 48,344 | 1,272 | 8,526 | 6,452 | 2,074 | 1,282 |
| Daejeon | **55,017** | 50,790 | 1,964 | 37,088 | 1,827 | 9,911 | 7,618 | 2,293 | 4,227 |
| Ulsan Metropolitan City | **70,157** | 69,331 | 1,818 | 60,587 | 987 | 5,939 | 4,060 | 1,879 | 826 |
| Sejong Special Self-Governing City | **8,025** | 7,912 | 335 | 5,689 | 428 | 1,460 | 1,009 | 451 | 113 |
| Gyeonggi-do | **527,273** | 506,840 | 18,896 | 408,782 | 13,390 | 65,772 | 45,882 | 19,890 | 20,433 |
| Gangwon-do | **119,311** | 112,491 | 4,470 | 89,008 | 3,522 | 15,491 | 11,251 | 4,240 | 6,820 |
| Chung-cheong bukdo | **120,307** | 113,962 | 11,814 | 85,684 | 1,625 | 14,839 | 11,195 | 3,644 | 6,345 |
| Chungcheongnam-do | **218,557** | 214,386 | 6,900 | 174,153 | 3,429 | 29904 | 22,609 | 7,295 | 4,171 |
| Jeollabuk do | **171,886** | 168,849 | 7,100 | 119,523 | 5,480 | 36,746 | 28,586 | 8,160 | 3,037 |
| Jeollanam-do | **217,543** | 205,493 | 10,192 | 157,084 | 3,729 | 34,488 | 24196 | 10,292 | 12,050 |
| Gyeongsangbuk-do | **239,895** | 229,180 | 8,985 | 178,102 | 4,603 | 37,490 | 27,016 | 10,474 | 10,715 |
| Gyeongsangnam-do | **300,078** | 295,302 | 13,472 | 234,695 | 4,916 | 42219 | 30,519 | 11,700 | 4,776 |
| Jeju Special Self-Governing Province | **35,142** | 34,131 | 2,124 | 24,544 | 1,351 | 6,112 | 5,044 | 1,068 | 1,011 |

Dementia Patient Dementia Relief Center Registration Status

▶ Source: Dementia Relief Center (2019)

▶ Extraction standard

1. Statistical base date ('~ '19.12.31) by using the ANSYS withdrawal function

| division | Total number of registered dementia patients | gender | | age | | | | | | | | | | |
| --- | --- | --- | --- | --- | --- | --- | --- | --- | --- | --- | --- | --- | --- | --- |
|  |  | **male** | **female** | **Less than 40 years** | **40-45** | **45-50** | **50-55** | **55-60** | **60-64** | **65-69** | **70-74** | **75-80** | **80-85** | **85+** |
| Nationwide | **421,012** | **117,302** | **303,710** | **20** | **42** | **110** | **429** | **1,467** | **5,655** | **14,964** | **33,352** | **78,116** | **117,667** | **169,190** |
| Seoul | **58,305** | 18,163 | 40,142 | One | 4 | 23 | 76 | 277 | 893 | 2,121 | 4,644 | 10,361 | 14,645 | 25,260 |
| Busan | **20,826** | 6,491 | 14,335 | One | 0 | 6 | 17 | 71 | 326 | 930 | 2,063 | 4,405 | 6,095 | 6,912 |
| Daegu Metropolitan City | **17,394** | 5,101 | 12,293 | 0 | 3 | One | 15 | 44 | 200 | 728 | 1,416 | 3,403 | 4,905 | 6,679 |
| Incheon Metropolitan City | **15,590** | 4,320 | 11,270 | 0 | 0 | 5 | 15 | 61 | 230 | 557 | 1,180 | 2,753 | 4,086 | 6,703 |
| Gwangju | **8,526** | 2,361 | 6,165 | 0 | 0 | 0 | 5 | 25 | 102 | 279 | 719 | 1,696 | 2,374 | 3,326 |
| Daejeon | **9,911** | 2,893 | 7,018 | 2 | 2 | 2 | 20 | 50 | 140 | 337 | 714 | 1,700 | 2,585 | 4,359 |
| Ulsan Metropolitan City | **5,939** | 1,643 | 4,296 | One | One | 0 | 11 | 42 | 154 | 347 | 556 | 1,163 | 1,580 | 2,084 |
| Sejong Special Self-Governing City | **1,460** | 421 | 1,039 | 0 | 0 | 0 | 0 | 5 | 18 | 52 | 117 | 303 | 390 | 575 |
| Gyeonggi-do | **65,772** | 19,481 | 46,291 | 5 | 10 | 21 | 70 | 266 | 1,027 | 2,443 | 5,262 | 11,970 | 18,404 | 26,294 |
| Gangwon-do | **15,492** | 4,281 | 11,211 | One | 2 | 7 | 25 | 71 | 250 | 520 | 1,099 | 2,741 | 4,391 | 6,385 |
| Chung-cheong bukdo | **14,839** | 4,290 | 10,549 | 0 | 2 | One | 17 | 63 | 234 | 502 | 1,019 | 2,608 | 4,251 | 6,142 |
| Chungcheongnam-do | **29904** | 8,597 | 21,307 | 2 | 5 | 7 | 11 | 78 | 333 | 945 | 2,246 | 5,333 | 8,815 | 12,129 |
| Jeollabuk do | **36,746** | 9,427 | 27,319 | One | 3 | 6 | 24 | 74 | 320 | 1,061 | 3,037 | 7,208 | 10,714 | 14,298 |
| Jeollanam-do | **34,488** | 8,202 | 26,286 | 0 | 2 | 5 | 22 | 72 | 325 | 1,031 | 2,762 | 6,956 | 10,421 | 12,892 |
| Gyeongsangbuk-do | **37,490** | 9,785 | 27,705 | 3 | 3 | 8 | 43 | 126 | 479 | 1,344 | 2,843 | 6,905 | 10,537 | 15,199 |
| Gyeongsangnam-do | **42,218** | 10,389 | 31,829 | 3 | 5 | 14 | 49 | 123 | 566 | 1,594 | 3,286 | 7,726 | 12,002 | 16,850 |
| Jeju Special Self-Governing Province | **6,112** | 1,457 | 4,655 | 0 | 0 | 4 | 9 | 19 | 58 | 173 | 389 | 885 | 1,472 | 3,103 |

( Unit: person )

 Current status of mild cognitive impairment registered at Dementia Relief Center

▶ Source : Dementia Relief Center (2019)

▶ Extraction standard

1) Excluding regressors

2) Total number of subjects registered in ANSYS on the statistical basis date ('~ Dec. 31, '19) who have mild cognitive impairment as a result of external diagnosis or who have mild cognitive impairment as a result of early screening at the Dementia Safety Center

| division | Cognitive impairment can be mild overall subscribers | gender | | age | | | | | | | | | | |
| --- | --- | --- | --- | --- | --- | --- | --- | --- | --- | --- | --- | --- | --- | --- |
|  |  | **male** | **female** | **Less than 40 years** | **40-45** | **45-50** | **50-55** | **55-60** | **60-64** | **65-69** | **70-74** | **75-80** | **80-85** | **85+** |
| Nationwide | **111,154** | **41,062** | **70,092** | **7** | **14** | **42** | **166** | **556** | **3,723** | **11,046** | **19,841** | **30,010** | **26,447** | **19,302** |
| Seoul | **53,438** | 20,313 | 33,125 | 5 | 11 | 25 | 95 | 297 | 1,426 | 4,675 | 9,479 | 14,276 | 12,694 | 10,455 |
| Busan | **5,718** | 2,126 | 3,592 | 0 | 0 | 2 | 3 | 23 | 248 | 685 | 1,165 | 1,711 | 1,242 | 639 |
| Daegu Metropolitan City | **3,251** | 1208 | 2,043 | 0 | 0 | One | 0 | 4 | 114 | 383 | 550 | 884 | 797 | 518 |
| Incheon Metropolitan City | **2,188** | 813 | 1,375 | 0 | 0 | One | 4 | 12 | 85 | 210 | 361 | 579 | 541 | 395 |
| Gwangju | **1,272** | 501 | 771 | 0 | 0 | 0 | 0 | 2 | 53 | 120 | 247 | 373 | 288 | 189 |
| Daejeon | **1,827** | 710 | 1,117 | 0 | 0 | 2 | 3 | 8 | 68 | 179 | 323 | 534 | 461 | 249 |
| Ulsan Metropolitan City | **987** | 396 | 591 | 0 | 0 | 2 | 5 | 14 | 86 | 177 | 191 | 240 | 184 | 88 |
| Sejong Special Self-Governing City | **428** | 153 | 275 | 0 | 0 | 0 | 0 | 0 | 10 | 52 | 72 | 115 | 94 | 85 |
| Gyeonggi-do | **13,390** | 5,167 | 8,223 | 0 | One | 3 | 12 | 54 | 574 | 1,400 | 2,435 | 3,507 | 3,190 | 2,214 |
| Gangwon-do | **3,522** | 1,209 | 2,313 | One | 0 | One | 4 | 21 | 115 | 286 | 505 | 986 | 975 | 628 |
| Chung-cheong bukdo | **1,625** | 578 | 1,047 | 0 | 0 | One | 5 | 11 | 67 | 193 | 250 | 433 | 412 | 253 |
| Chungcheongnam-do | **3,429** | 1,252 | 2,177 | One | 0 | 0 | 3 | 10 | 156 | 366 | 605 | 860 | 867 | 561 |
| Jeollabuk do | **5,480** | 1,821 | 3,659 | 0 | 0 | 0 | 7 | 13 | 220 | 689 | 1,082 | 1,564 | 1,209 | 696 |
| Jeollanam-do | **3,729** | 1,217 | 2,512 | 0 | 0 | 0 | 4 | 10 | 105 | 334 | 588 | 1,133 | 930 | 625 |
| Gyeongsangbuk-do | **4,603** | 1,521 | 3,082 | 0 | 0 | One | 9 | 33 | 167 | 596 | 816 | 1,176 | 1,084 | 721 |
| Gyeongsangnam-do | **4,916** | 1,616 | 3,300 | 0 | 2 | 3 | 11 | 33 | 189 | 593 | 974 | 1,305 | 1,143 | 663 |
| Jeju Special Self-Governing Province | **1,351** | 461 | 890 | 0 | 0 | 0 | One | 11 | 40 | 108 | 198 | 334 | 336 | 323 |

( Unit: person )

AchE + Memantine or Choline alfoscerate

| Dementia Medication Prescription Status_ Nursing Days |
| --- |
| Source: National Health Insurance Corporation (2019)  Substitution Criteria:  1) Excluding non-payment. Excluding deaths, including overlapping by type of medical institution. 2) A person with dementia has been diagnosed with six dementia disease codes (F00, F01, F02, F03, G30, G31) based on ICD-10 codes.  3) Dementia care: Hospitalization, outpatient and pharmaceutical prescriptions with dementia codes |

| ※ Ingredient combination parallel | |
| --- | --- |
| D: Donepezil | 148601ATB','148601ATD','148602ATB','148602ATD','148603ATB' |
| G: Galantamine | 385203ACR','385203ATR','385204ACR','385204ATR','385205ACR','385205ATR' |
| R: Rivastigmine | 224501ACH','224503ACH','224504ACH','224505ACH','224506CPC','224507CPC' |
| M: Memantine | 190001ALQ','190001ATB','190001BIJ','190002ASY' |
| C: Choline alfoscerate | 138101ACH','138101ACS','138101APD','138101ATB','138102BIJ' |

| D | 56174057 |  |
| --- | --- | --- |
| G | 1967039 |  |
| R | 2364961 |  |
| M | 7791481 |  |
| C | 23914113 |  |
| D+M | 7892440 |  |
| G+M | 313882 |  |
| R+M | 563054 |  |
| D+C | 19418212 |  |
| G+C | 955319 |  |
| R+C | 1130814 |  |
| D+M+C | 3988509 |  |
| G+M+C | 183702 |  |
| R+M+C | 283490 |  |
| ETC | 10350168 |  |

ETC

| Serial No. | 1 | 3 | 4 | 5 | 6 | 7 | 8 | 9 | 10 |
| --- | --- | --- | --- | --- | --- | --- | --- | --- | --- |
| Ingredient name corresponding to ETC | quetiapine fumarate | magnesium hydroxide | escitalopram oxalate(as escitalopram) | mosapride citrate | alprazolam | rebamipide | clopidogrel | aspirin(enteric coated) | clonazepam |

**References**

1. Jong Hoon L, Badar K, Chul Joong L, Consolato S, Michael C. Dapsone is an anticatalysis for Alzheimer’s disease exacerbation. Research Square. 2022. doi: 10.21203/rs.3.rs-1260878/v1.

2. Oldenburg CE, Pinsky BA, Brogdon J, Chen C, Ruder K, Zhong L, et al. Effect of Oral Azithromycin vs Placebo on COVID-19 Symptoms in Outpatients With SARS-CoV-2 Infection: A Randomized Clinical Trial. JAMA. 2021. doi: 10.1001/jama.2021.11517.
